# Actions against the double burden of malnutrition in Peru: a community-informed system dynamics model

**DOI:** 10.1016/j.lana.2025.101102

**Published:** 2025-04-21

**Authors:** Paraskevi Seferidi, Laura Guzman-Abello, Ellis Ballard, Hilary M. Creed-Kanashiro, Luis Huicho, J Jaime Miranda, Christopher Millett, Antonio Bernabe-Ortiz

**Affiliations:** a Public Health Policy Evaluation Unit, School of Public Health, Imperial College London, London, UK; b Department of Design, School of Architecture and Design, Universidad de los Andes, Bogota, Colombia; c Social System Design Lab, Brown School at Washington University in St. Louis, St. Louis, USA; d Instituto de Investigación Nutricional, Lima, Peru; e Centro de Investigación en Salud Materna e Infantil, Centro de Investigación para el Desarrollo Integral y Sostenible and Facultad de Medicina, Universidad Peruana Cayetano Heredia, Lima, Peru; f CRONICAS Centre of Excellence in Chronic Diseases, Universidad Peruana Cayetano Heredia, Lima, Peru; g Sydney School of Public Health, Faculty of Medicine and Health, University of Sydney, Sydney, Australia; h NOVA National School of Public Health, Public Health Research Centre, Comprehensive Health Research Center, NOVA University Lisbon, Lisbon, Portugal

**Keywords:** Double burden of malnutrition, Double-duty actions, Peru, System dynamics

## Abstract

**Background:**

Peru's progress in reducing stunting has stagnated since 2018, while the country is facing increasing levels of overnutrition, leading to a double burden of malnutrition. However, this shift in nutrition burden is not reflected in Peru's nutrition policy agenda. This study aims to identify leverage points for actions against population-level double burden of malnutrition in Peru.

**Methods:**

We developed a system dynamics model that simulates changes in overweight and stunting over time in Peru through changes in food system drivers. The model was conceptually informed by policymakers, practitioners and community members in Peru and used quantitative and qualitative data from secondary sources and published literature.

**Findings:**

The model indicated that several overnutrition policies, including policies targeting food availability and affordability, may decelerate but not halt the increase of overweight in the country, mainly due to industry resistance. However, in the long term, the reallocation of resources towards overnutrition policies may inadvertently hinder progress towards stunting targets. Transforming nutrition policy governance, from siloed overnutrition and undernutrition policies towards a common policy framework against the double burden of malnutrition was the only modelled scenario that halted the rise in overnutrition, while keeping Peru on course to reach its stunting goals.

**Interpretation:**

Transition away from policy landscapes that focus on single nutrition outcomes towards synergistic actions that target malnutrition in all forms is a long-term solution towards achieving global nutrition goals. Such policy transitions are especially important in low and middle-income countries like Peru, which are affected by the double burden of malnutrition.

**Funding:**

This study was supported by a research grant from the 10.13039/501100000268Biotechnology and Biological Sciences Research Council (BBSRC) (grant reference: BB/T009004/1).

**Research in context**

**Evidence before this study**

We search PubMed and Latin American and Caribbean Health Sciences Literature (LILACS) databases for studies on the double burden of malnutrition in Peru, using the terms “double burden of malnutrition” and “Peru” with no time or language restrictions. Most existing literature includes epidemiological studies that describe double burden of malnutrition trends or associations with covariates. One qualitative study assessed the implementation and prioritisation of policies against the double burden of malnutrition in infants and young children in Peru using interviews with experts. One non-peer reviewed modelling study, published by Peru's Ministry of Health, explored the social and economic burden of the double burden of malnutrition in the country. However, no previous study has modelled the potential impact of ‘what-if’ scenarios on double burden of malnutrition in Peru.

**Added value of this study**

This study uses a conceptual system dynamics model to simulate the impact of different ‘what-if’ scenarios against the double burden of malnutrition in Peru. It benefits from the active involvement of stakeholders in the model conceptualisation and the consideration of the food system complexity that drives the double burden of malnutrition. It identifies intervention points that have the leverage to improve population-level double burden of malnutrition in Peru, defined as the co-existence of overweight and stunting.

**Implications of all the available evidence**

To achieve long term reduction in both overweight and stunting in Peru, there needs to be a transformation of existing siloed decision-making approaches that consider overnutrition and undernutrition as two separate issues towards common policy efforts against malnutrition in all its forms.

## Introduction

Recognising the urgent need to address malnutrition in all its forms and achieve the Sustainable Development Goals, the World Health Organization (WHO) has set its 2025 Global Nutrition Goals, which include a reduction in under-5 stunting prevalence by 40% and no further increase in childhood overweight and obesity.^1^ Peru has been described as a success story in its efforts to achieve its stunting goals,^2^ with the stunting rate decreasing from 31.3% in 2000 to 11.5% in 2021.^3^ This was significantly driven by strong civil society advocacy and keen political leadership for a multisectoral approach to prioritise stunting reduction in the national political agenda.^4^ At the same time, Peru has recently implemented some bold policies against overweight and obesity, such as warning labels and marketing restrictions for processed foods high in sugar, fats, and sodium, especially in schools.^5^ These efforts attempt to tackle the persistent increase of overweight and obesity of the last 20 years, with 63% of adults^6^ and 9.7% of children under-5^7^ being overweight or obese in 2021.

Peru's overnutrition policy efforts align well with policy recommendations by the WHO^8^ and other influential policy frameworks^9^^,^^10^ to target unhealthy food marketing, labelling, and promotion. However, such actions tend to focus on isolated components of the food system, often ignoring potential unintended side-effects.^11^ This is especially important in contexts, such as Peru, where the nutrition transition has led to the co-existence of overnutrition and undernutrition at national level, which is often referred to as double burden of malnutrition (DBM).^12^ Despite this co-existence, governance of overnutrition and undernutrition policy is often siloed. For example, in Peru, overnutrition policy, such as front of pack warning labels, is led by the Ministry of Health, whereas policies that target undernutrition, such as food assistance and conditional cash transfer programmes, are led by the Ministry of Development and Social Inclusion. This misalignment between nutrition policy governance and priorities and the emergence of DBM may unintendedly result in ineffective policies due to a lack of interdisciplinary capacity and coordinated leadership and commitment.^13^

In response to the increasing levels of DBM globally, WHO has called for double-duty policies. These include policies that target one type of malnutrition while ensuring that they have no unintended consequences to other types of malnutrition, as well as policies that concurrently address several types of malnutrition.^14^ However, implementation of double-duty policies remains limited.^15^ Although opportunities for double-duty actions have been previously recommended,^14^^,^^16^ these are broad in nature and do not consider local food systems and views of local stakeholders. In previous work, we used inputs from local stakeholders from two diverse regions in Peru to qualitatively characterise the food system drivers of the DBM in Peru from a feedback perspective.^17^ In this study, we used these inputs to inform a system dynamics (SD) simulation model that aims to identify opportunities for actions that can address the population-level DBM in Peru, defined here as the co-existence of overweight and stunting at the national level.

## Methods

### Overview of system dynamics modelling

The need for systems-informed approaches has been increasingly emphasised to improve understanding of the food system and related issues, such as the DBM.^18^, ^19^, ^20^ SD is a systems-informed simulation modelling approach that can be used to understand complex, dynamic problems. SD can explain how a problem may change in response to the different ways that components of a complex system interact with each other over time,^21^ often resulting in feedback loops. Further description of main SD concepts is presented in Supplementary Text S1.

There are several reasons why SD was considered the most appropriate approach for this study. First, the design and implementation of double-duty actions require integration of diverse governance and capacity strengthening mechanisms that may have previously acted in isolation.^22^ SD provides an opportunity to explore interactions across several components of the system, encouraging views of diverse stakeholders to emerge. Using qualitative systems mapping to inform model structure in this study can allow diverse perspectives to come together, including policymakers and health practitioners, food system experts, and community members with lived experiences within the local food system. Second, double-duty actions pertain to the prevention of unintended harm,^15^^,^^22^ as they ought to ensure that actions targeting one type of malnutrition have no unintended side effects on other forms of malnutrition. A SD approach can reveal unintended or unanticipated side effects of policies, as it moves away from interpreting observed behaviour as the result of independent external factors and recognises that the way these factors interact over time can lead to unexpected outcomes.^23^ Finally, this study did not intend to produce explicit forecasts of DBM epidemiology, but rather to characterise how DBM patterns of behaviour may change when the dynamics that drive them change. This fits well with SD's scope, which is to enable characterisation of plausible scenarios under changes that occur within a complex system.^24^

### Overview of modelling process

**Fig. 1:**
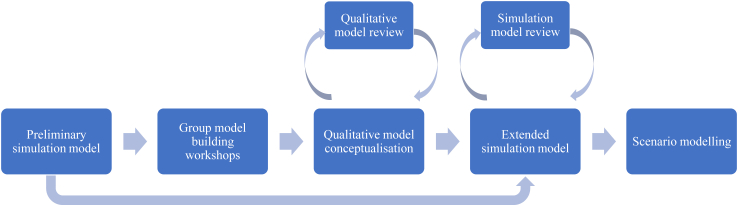
Overview of modelling process.

We developed a SD simulation model to understand changes in overweight and stunting/short stature in children and adults of reproductive age in the overall Peruvian population over time. The model was developed in Vensim DSS Version 10.1.3. An overview of the modelling process is presented in Fig. 1 and described in more detail below.

#### Preliminary simulation model

We developed a preliminary stock and flow structure based on standard formulations of population dynamics as described by Sterman^25^ and epidemiological data on population demographics and prevalence of overweight and stunting in Peru from Peru's Demographic and Health Surveys, Peru's National Institute of Statistics and Informatics (Instituto Nacional de Estadística e Informática), and the World Bank. In the model, we defined overweight, which includes both overweight and obesity, as weight for height z-score >2 SD, BMI-age z-score >1 SD, and BMI >25 kg/m^2^ for children under-5, children 5–19, and adults respectively. We defined stunting in children as height for age z-score <−2 SD. The stock and flow structure comprises three aging chains that represent: (1) the overall Peruvian population; (2) the stunted/short stature Peruvian population, and (3) the overweight Peruvian population, from birth until end of adulthood. It also captures the intergenerational transmission of stunting through maternal short stature and low birth weight.^26^ The structure and data sources used in this component of the model are presented in Supplementary Table S1.

#### Group model building workshops and qualitative model conceptualisation

The model conceptualisation was based on inputs from Peruvian stakeholders. Details on the design, implementation, and outputs of stakeholder workshops are presented elsewhere.^17^ Briefly, we conducted five in-person group model building workshops in April 2022 with diverse stakeholders from two regions in Peru: Lima, the country's capital city with one of the highest rates of overweight in the country, and Iquitos, a large city in the Amazonian jungle with high levels of both stunting and overweight prevalence and presence of indigenous communities. As the Peruvian health and nutrition decision making system is mostly centralised, participants in the Lima workshops were able to offer an insight into national issues and policies. Additionally, the inclusion of stakeholders from Iquitos allowed participation of stakeholders with an understanding of the system that drives the DBM at a regional level. Perspectives from all stakeholders were synthesised into a single conceptual model, with no explicit differentiation between regions, due to lack of region-specific data.

We followed a purposive sampling approach to recruit stakeholders with decision-making power and who are knowledgeable about the problem, while ensuring a wide variety of viewpoints, as previously recommended.^27^ Our final sample included policymakers from various government departments that have been involved in nutrition policy decision making (n = 9), health practitioners and academics with a deep understanding of the Peruvian food system (n = 9), representatives of NGOs with presence across the country (n = 7), and mothers of young children of diverse socioeconomic status and with lived experiences within the local food system (n = 11), with a total of 23 participants in Lima and 13 in Iquitos. Further details of workshop participants have been detailed elsewhere.^17^

The main aim of the workshops was to characterise food system drivers of the DBM according to stakeholders’ experiences. We used the HPLE food systems conceptual framework^28^ to define the boundary of our model and guide model development. The main output of stakeholder workshops was a causal loop diagram, which was used as the basic conceptual formulation of the simulation model that describes the baseline scenario in this analysis. An online review session with a subgroup of workshop participants was also conducted to refine this causal loop diagram.^17^

#### Extended simulation model

We used information from the causal loop diagram developed by stakeholders to extend our preliminary simulation model. The model formulation process required the addition of structures and exogenous variables to effectively operationalise qualitative feedback loops, described by stakeholders during workshops. The model was quantified using data from various secondary sources, prioritising relevance to the Peruvian population. Effect estimates were obtained from published literature, prioritising studies from Peru and where these were not available, studies from other settings in Latin America or pooled estimates from systematic reviews and meta-analyses. Food system variables, including diet and food industry estimates, were Peru-specific and obtained from local and international sources, such as the Global Dietary Database and Peru's National Household Survey (ENAHO). Finally, when specific data were not available, relevant assumptions were made, mostly informed by published literature related to the policy landscape in Peru. All data inputs, their sources, and assumptions are presented in detail in Supplementary Table S1. An online model review session was conducted in June 2023 to test some of these assumptions and confirm updated model structures. A total of six stakeholders from Lima participated in this session. This model was used to simulate the baseline scenario, which describes the dynamic behaviours that have historically driven overweight and stunting in Peru.

We performed several tests to build confidence in the model, as suggested by Sterman.^25^ First, we tested the ability of the model to reproduce the behaviour of the system by visually comparing simulated adult overweight, under-5 stunting, total adult population, and total child population behaviour-over-time graphs with observed trends between 2000 and 2019 in Peru, using overweight and stunting data from Peru's Demographic and Health surveys and population data from Peru's National Institute of Statistics and Informatics (Supplementary Text S2). We chose these variables due to the availability of observed data that allowed comparison with observed trends. Second, we ensured that all constants and variables in the model were well-defined and their units of measurement were consistent, while we also clearly described all the assumptions made and data sources used (Supplementary Table S1). Third, as system dynamics models use differential equations solved by numerical integration, we explored the impact of different integration methods and time steps on adult overweight and under-5 stunting (Supplementary Text S3). We also performed sensitivity analyses by varying each constant variable, i.e. variable that does not change over time, by ± 10%. This included running the model multiple times, each time with a different constant variable variation, using the Sensitivity2All tool in Vensim. We, then, reported how these variations impacted simulated over-time behaviour of adult overweight and under-5 stunting prevalence (Supplementary File S4). Finally, we tested different extreme scenarios to explore if the model produced expected behaviours under these conditions (Supplementary Text S5).

#### Scenario modelling

Following the simulation of the baseline scenario and drawing from insights from stakeholder workshops, the research team developed different ‘what-if’ scenarios to explore options for changing the patterns of behaviour of overweight and stunting. Scenarios were developed across different leverage points based on the Donella Meadows' framework of the 12 places to intervene in a system.^29^^,^^30^ Meadows' leverage points framework identifies different levels of intervention within a complex system that can lead to a system change at increasing levels of effectiveness. These levels of intervention range from isolated actions, with small potential to achieve long term changes, to actions that transform the structures, mindsets and paradigms of the whole system, with the power to effectively transform complex problems. We developed ‘what-if’ scenarios across five leverage points informed by the Meadow's framework, which included: (*1*) parameters, i.e. changes to isolated actions, such as taxes and subsidies; (*2*) delays, such as alignment of timeframes in policy and decision making processes; (*3*) feedback loops, such as actions that balance mechanisms that drive a problematic behaviour or reinforce actions that drive a problem towards the right direction; (*4*) system rules, such as changes in the ways that the system is regulated; (*5*) system goals, such as changes in the goals that drive decisions.^29^^,^^30^ The specific leverage points modelled were informed by existing nutrition policy frameworks,^9^^,^^10^ WHO recommendations for double-duty actions,^14^ and previous literature on actions against the DBM in Peru,^31^^,^^32^ triangulated with actions that emerged from the model structure, such as its delays and feedback mechanisms.

### Ethical approval

Stakeholder workshops that informed model conceptualisation have received ethical approval by the Institutional Research Ethics Committee of Universidad Peruana Cayetano Heredia (approval number: 104098).

### Role of the funding source

The funder of the study had no role in study design, data collection, data analysis, data interpretation, or writing of this report.

## Results

### Baseline scenario

**Fig. 2:**
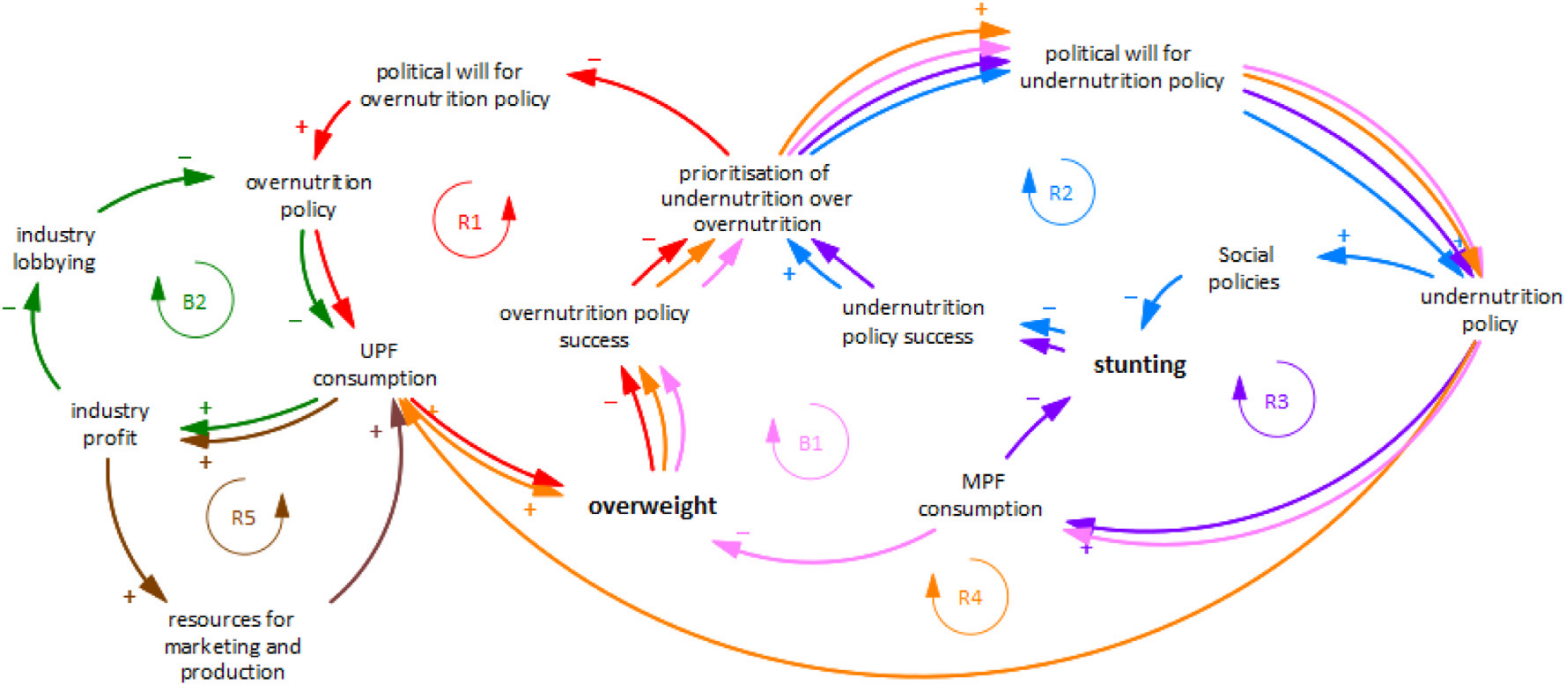
Causal loop diagram of baseline scenario.

The baseline scenario assumed in this study, as informed by relevant stakeholders, is presented as a causal loop diagram in Fig. 2. The causal loop diagram comprised five reinforcing (R) feedback loops and one balancing (B) feedback loop (Supplementary Table S2, Fig. 2). In this baseline scenario, undernutrition policy targets stunting through social and economic policies to alleviate poverty (R2 in Fig. 2) and through food provision policies, which directly impact food consumption. These policies can provide both healthy foods, here represented as minimally processed foods (MPF) (R3 in Fig. 2), and unhealthy foods, here represented as ultra-processed foods (UPF) (R4 in Fig. 2). According to stakeholders, due to the historic success of undernutrition policy over the years, undernutrition efforts have been and remain prioritised over overnutrition in Peru's nutrition policy agenda. This historic prioritisation of undernutrition policies, beyond successfully reducing stunting, can also lead to less political will for overnutrition policies, diluting policy success against overnutrition (R1 in Fig. 2). In this baseline scenario, we assumed that overnutrition policy targets consumption of unhealthy foods, such as UPF, by reducing UPF acceptability, through nutrition education and marketing restrictions, and UPF availability. At the same time, UPF consumption may be driven by food industry marketing and overproduction, ultimately aiming to increase industry profits (R5 in Fig. 2). Industry also acts by resisting overnutrition policy through lobbying (B1 in Fig. 2).

Under this baseline scenario, the model demonstrated a S-shaped increase over time in overweight among adults of reproductive age and a reduction in under-5 stunting, which appeared to be more rapid during the second half of the 2000s decade and has stabilised over recent years. These simulated behaviours generally align with observed overweight and stunting trends between 2000 and 2019 in Peru (Supplementary Text S2). Furthermore, sensitivity analyses indicated that varying constant variables in the model did not substantially alter simulated behavioural patterns of adult overweight and under-5 stunting (Supplementary Text S4).

### Scenario modelling

Modelled scenarios are presented in Table 1. They were assumed to initiate in 2015, which approximates the time when overnutrition policy started to be implemented in Peru. Each scenario is modelled in addition to all previous scenarios. We further clarify the assumptions made under each scenario on Supplementary Table S3.

**Table 1:** Modelling scenarios.

Leverage point	Scenario	Scenario description
Parameters	S1	Introduction of price policies
Delays	S2	S1 + Reduce policy delay, i.e. the time between political will and policy action for both overnutrition and undernutrition
Feedback loops	S3	S2 + Increase overweight monitoring: Overnutrition policy success is as well monitored as undernutrition policy success
Rules of the system	S4	S3 + Regulate quality of foods available through undernutrition policies: No ultra-processed foods can be provided through undernutrition policies, such as food assistance programmes
	S5	S4 + Regulate industry lobbying: Industry plays no role in the overnutrition policy making process
Goals of the system	S6	S5 + Double-duty policies informed by malnutrition in all its forms progress

Note: Each Scenario includes all the previous scenarios.

**Fig. 3:**
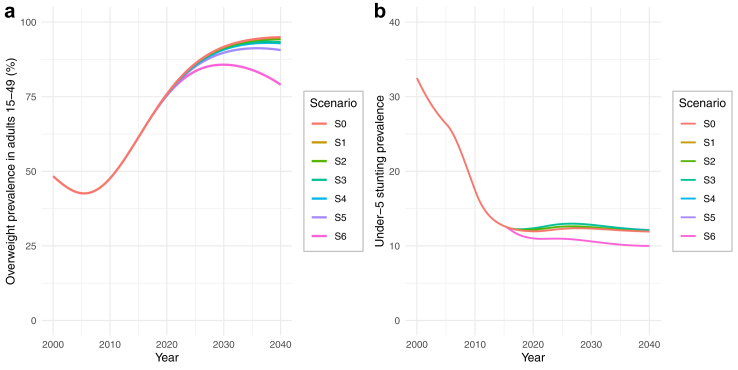
Behaviour over-time graphs for (a) adult overweight prevalence and (b) under-5 stunting, under different scenarios.

*Scenario 1* represents changes in the parameters of a system, the least effective intervention point according to the Meadow's framework. In this scenario, overnutrition policy in Peru is further extended to incorporate an increase in the price of UPF, for example through the implementation of a tax on UPF. The model indicates that such changes are unlikely to achieve any notable reductions in UPF consumption (Supplementary Figure S1) or in overweight (Fig. 3a), given the system's resistance to change. Specifically, reductions in UPF consumption would lead to an increase in industry efforts to increase production and marketing of UPF (Fig. 2, R5), balancing out policy actions and preventing any notable impacts.

*Scenario 2* intervenes in the delays of the system. We simulated the potential impact of reducing the delay between political will for policy change and policy implementation for both overnutrition and undernutrition policy, for example through streamlining decision-making processes by improving intragovernmental communication and strengthening the advocacy role of organised civil society. Under this scenario, a faster increase in overnutrition policy efforts is observed. However, given that feedback mechanisms that drive UPF consumption and resist overnutrition policy (Fig. 2, R5 and B1) still persist under this scenario, only a slight reduction in UPF is observed (Supplementary Figure S1), which may not be sufficient to alter how overweight changes over time (Fig. 3a).

*Scenario 3* intervenes to increase the gains of the reinforcing feedback loop R1 by improving monitoring efforts for overweight and achieving the same monitoring capability evident for undernutrition. Stunting in children under-5 in Peru is monitored annually at national and regional level through well-established data collection processes. These estimates are used to directly inform progress against stunting at regional level. This scenario assumes that existing undernutrition monitoring processes are expanded to include overweight prevalence for adults as a success measure of overnutrition policy. Although this may increase political will and thus policy efforts against overweight (Fig. 2, R1), resulting in a slight reduction in UPF in the short-term (Supplementary Figure S1), it will also ramp up industry efforts that counterbalance this initial success (Fig. 2, R5 and B1). Thus, this reduction may not be maintained in the long term to meaningfully change overweight patterns of behaviour (Fig. 3a).

*Scenarios 4* and *5* aim to change the rules of the system by removing the reinforcing feedback originating from provision of UPF by undernutrition food assistance programmes (*Scenario 4*) and removing the balancing feedback resulting from industry's lobbying efforts to delay policy action (*Scenario 5*). More specifically, Scenario 4 assumes that food assistance programmes eliminate the provision of UPF, for example through compliance to dietary quality standards for food assistance programmes to exclude UPF. Scenario 5 assumes elimination of policy delays due to industry lobbying, which can be achieved through restrictions in interactions between industry and policymakers and establishment of absolute transparency in all industry interactions, informed by existing regulations for restriction of tobacco industry interference, such as those established by the WHO Framework Convention on Tobacco Control.^33^ Our model shows that Scenario 4 has a negligible impact on consumption of UPF and related overweight, given the small percentage of UPF that can be attributed to food assistance programmes, compared to the overall diet. However, Scenario 5 manages to ramp up overnutrition policy efforts and advance UPF reduction (Supplementary Figure S1) mainly because it acts against an important industry resistance mechanism (Fig. 2, B1). However, given that alternative industry resistance mechanisms are maintained (Fig. 2, R5), the corresponding reduction in overweight do not result in a reversal to the baseline increase in overweight over the years (Fig. 3a).

Although some of the scenarios modelled above may manage to improve policy efforts against overnutrition, they may also result in unintended consequences to undernutrition policy efforts. This is because of the system's structure to prioritise successful policies for one type of malnutrition against the success of policies for other types of malnutrition (Fig. 2, R1 and R2). According to stakeholders, this may have historically resulted in a prioritisation of undernutrition over overnutrition policy, due to the former's policy success over the years. Similarly, however, any potential future success of overnutrition policies may result in less political will and resources being available for undernutrition policies, hindering or even reversing further progress towards stunting goals (Fig. 3b).

**Fig. 4:**
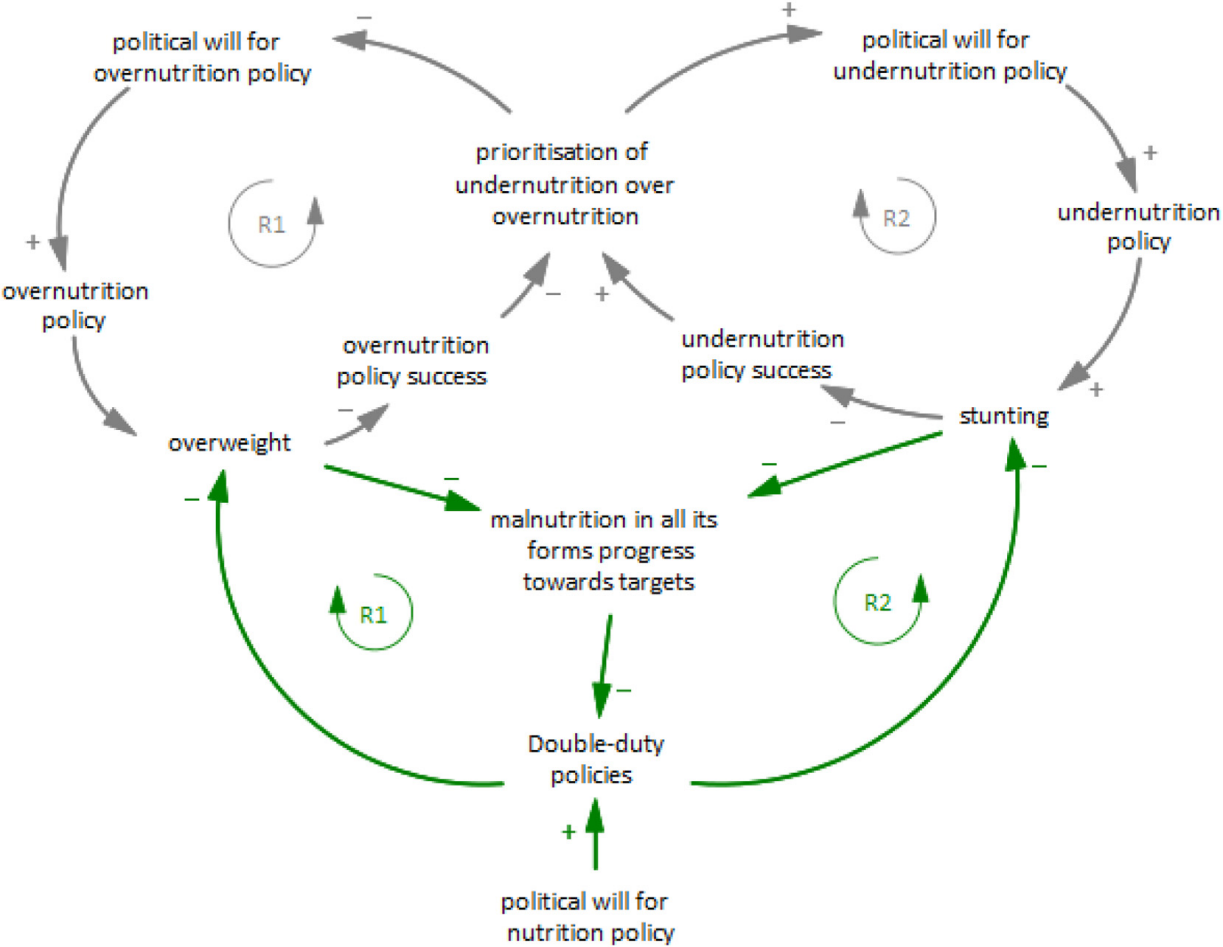
Causal loop diagram showing a system shift from prioritisation of undernutrition over overnutrition (Baseline scenario, grey lines) to double-duty actions (Scenario S6, green lines).

In *Scenario 6*, we modelled an opportunity to overcome this issue by changing the goals of the system, which is one of the most effective leverage points according to the Meadows framework. Scenario 6 models a transformation away from a system where resources and political will for nutrition action are competitively divided between overnutrition and undernutrition policy (Fig. 4, R1a and R2a). Instead, progress against targets for both overnutrition and undernutrition drives resource allocation and political will for nutrition action against the DBM (Fig. 4, R1b and R2b). Under this scenario, political will for nutrition action increases until goals for both overweight and stunting are met, allowing for an increase and long-term continuity of both overnutrition and undernutrition policies. We refer to policies under Scenario 6 as double duty policies. In this Scenario, double duty policies do not include a change in the way they act against overweight and stunting, but a change in the way they are governed, regulated, and monitored; from siloed overnutrition and undernutrition policies towards a common policy framework against the DBM. Under Scenario 6, the model showed a halt and even a reversal in the long-term increase of overweight (Fig. 3a). Importantly, the dedicated political will against malnutrition in all its forms may stimulate a further decrease in stunting, contrary to the plateaued behaviour shown at baseline (Fig. 3b).

## Discussion

This study used a conceptual system dynamics model to simulate the food system dynamics driving the behaviour of overweight and stunting over time in children and adults of reproductive age at the national level in Peru. The model was subsequently used to identify intervention points in the food system that can improve the double burden of overweight and stunting in the country. We found that efforts to reverse the increasing levels of overweight in Peru are likely to fail in the long term due to the system's resistance to change, mainly attributed to food industry resistance to policy and the historical prioritisation of undernutrition in the country's policy agenda. Notably, any policy success against overweight is likely to unintentionally dilute policy efforts against stunting, due to policy structures that treat different types of malnutrition as separate issues. According to this model, to achieve long-term reduction in both overweight and stunting, Peru needs a transformation of existing siloed decision-making approaches towards common policy efforts against malnutrition in all its forms.

This paper presents recommendations on how double-duty policies should be governed and monitored to effectively target malnutrition in all its forms in Peru. This complements existing research about priority double-duty actions in the country. For example, previous research engaging with national experts in Peru has supported the need to boost political support, set evidence-based goals, and secure dedicated funding explicitly for the DBM, to effectively reduce DBM in infants and young children.^31^ Our study quantitatively confirms the requirement of such changes for double-duty policies to have a long-term impact against overweight and stunting. The need for coordinated governance for all forms of malnutrition has also been recognised as a priority at a global level,^16^ with Hawkes and colleagues recommending that to achieve this shift in nutrition policy governance, a change in nutrition policy funding must first occur, as undernutrition policies still dominate nutrition budgets in most LMICs.^16^ This may require funding support from diverse government departments, shifting the funding responsibilities from the health sector alone. Changes in funding support should also coincide with efforts to shift current knowledge, beliefs, and mindsets among policymakers, implementors and evaluators, recognising the need for a common approach against malnutrition in all its forms.^16^ Finally, coordination across sectors and government departments, which is necessary to achieve effective double-duty actions, requires strong capacity building for key policy stakeholders and institutions, as highlighted by the Global Sustainable Development Report.^34^ Peru is a highly diverse country with varied DBM levels and nutritional priorities across its departments. This diversity, in combination with often inadequate efforts to decentralise policy governance, highlights the need for capacity building at multiple governance levels, including support for congressional dialogue at national level, developing technical capacity at local levels, and improving accountability mechanisms at both levels.

Our model indicates the importance of achieving a system transformation towards coordinated actions against the double burden of malnutrition in Peru. Practical recommendations on how to achieve this can be drawn from Peru's stunting success story, which was largely driven by bold transformations of governance structure. First, Peru has previously demonstrated successful coordination between various government departments in its efforts to reduce stunting.^31^ For example, the establishment of the Ministry of Development and Social Inclusion in 2011 explicitly aimed to achieve coordination between social and nutrition policies. It oversaw several social and antipoverty policies that for the first time integrated nutrition-specific targets.^35^ This integration of social and nutrition policy significantly contributed to the success of programmes, such as the cash assistance programme JUNTOS, in reducing stunting in Peru.^4^ Similar coordination actions that concentrate nutrition efforts against both overnutrition and undernutrition under a commonly co-ordinated ministerial department have the potential to benefit Peru's nutrition governance. Second, Peru used an innovative approach to financing nutrition programmes, following a Results-Based Budgeting model, where budgeting was determined based on regional progress in reaching stunting reduction goals. This transparent and cross-cutting approach to financing led to implementation of nutrition programmes that benefited from commitment by local policymakers and support from the public^36^ and has the potential to be expanded to account for progress against malnutrition in all its forms. Finally, civil society played a significant role in Peru's stunting reduction success. Their concerted advocacy efforts influenced political decisions to set specific reduction targets, commit to funding allocation for undernutrition programmes, and develop the Ministry of Development and Social Inclusion to oversee progress.^4^ Similar engagement of active civil society advocacy for policy against malnutrition in all its forms is crucial in order to achieve change in current decision-making processes. These recommendations are further corroborated by the success of Brazil's nutritional policy. Brazil’ nutrition governance is deeply tied to the National Council on Food and Nutrition Security, a multisectoral body with direct support from Brazil's President and a strong civil society presence. Explicit nutrition targets are used to inform public budget distribution, while decision making is heavily influenced by civil society and citizen participation through a quadrennial national conference.^37^ As Peru's nutrition burden shifts towards malnutrition in all its forms, learnings from both within the country and other successful cases in the region can be used as a springboard towards successful policy transformation.

This paper used a conceptual system dynamics simulation model that was grounded in inputs from diverse local stakeholders. The model can be used as a tool to visualise the system as a whole, build a shared language, and develop a systems thinking understanding of double burden of malnutrition drivers in Peru among policymakers, researchers, and advocates. It can also give these actors agency around the double burden of malnutrition problem and invite them to see themselves as part of the system. It can test our assumptions about existing policy actions and identify broad opportunities for effective leverage point interventions by contributing to the understanding of their potential expected and unexpected consequences. However, the model should not be misused as a tool to make forecasts about future overweight and stunting prevalence, predict the future impact of specific policies, or inform policy planning decisions, e.g. for resource allocation or policy operationalisation.

This model has benefited from the active involvement of stakeholders in its conceptualisation, has shown robustness across various confidence building tests and has revealed important policy resistance mechanisms against double-duty actions in Peru. However, it also has several limitations. First, there was a lack of quantitative data related to the food industry market mechanisms and responses. Moreover, workshop participants did not include food industry stakeholders. This further minimised our understanding of food industry responses, which was mainly informed by inputs from policymakers, researchers, and NGO representatives, and may have resulted in a misestimation of policy resistance in our model, which was largely attributed to industry response to policy. Future model development should further explore the role of food industry on diet intake in Peru, including by engaging with industry stakeholders where appropriate, as well as stakeholders from consumer organisations and other advocacy groups with a deep understanding of industry operation and lobbying. Second, sensitivity analyses demonstrated that the model is sensitive to changes in some qualitative variables, such as industry goals for UPF consumption and political will for nutrition policy. Further consolidation of the quantification of these variables can improve future iterations of the model. Third, the model focused on reducing consumption of unhealthy foods, such as UPF, as the main policy mechanism to reduce overweight. This reflects the mechanisms of existing policy regulations, such as warning labels and advertising restrictions. However, overnutrition policy can further be targeted through several other health and socioeconomic mechanisms, such as access to healthcare, financial circumstances and physical activity behaviours. Future modelling scenarios can explore opportunities for multi-sectoral overnutrition interventions that consider a wider range of drivers. Fourth, although the model conceptualisation was informed by stakeholders from two diverse regions in Peru, Lima and Iquitos, the simulation model is aggregated at national level. This has limited the model's capacity to capture region-specific dynamics, including region-specific unintended side effects of undernutrition policies suggested by stakeholders in Iquitos.^17^ Future model iterations should incorporate local-level dynamics of nutrition policy to simulate differential impacts across regions, obtaining further inputs from diverse stakeholders, including from rural communities and Peru's highland regions. Fifth, data used to test the ability of the model to reproduce observed behaviour were collected before 2020, which impacts our ability to assess behaviour reproduction after that. Finally, the model relies on several assumptions, fully described in Supplementary Table S2. Although efforts have been made to test these assumptions by triangulating with observed data and stakeholder inputs, and calibrating the model accordingly, these assumptions should be taken into account when interpreting the results of this paper. Modelling complex social systems faces a significant challenge due to the lack of appropriate data to measure this complexity in a standardised way. By making our assumptions, model structure, parameter estimates, and purpose of our model transparent, we acknowledge and engage with this complexity, without attempting to provide a perfect representation of reality, while allowing future modellers to refine and enhance the model as further empirical data become available.

Our model shows that the success of efforts to address the double burden of malnutrition in Peru may be hindered if nutrition policy governance, monitoring and evaluation does not shift from a siloed approach that considers overnutrition and undernutrition as two separate issues into an integrated approach that targets malnutrition in all its forms. Peru's stunting reduction success should not act as hindrance to political will for overnutrition policy, through the prioritisation of successful undernutrition policies in the expense of overnutrition efforts. It can instead act as a paradigm in Peru's efforts for cross-government coordination and civil society advocacy to tackle malnutrition in all its forms.

## Contributors

PS, CM, and ABO conceptualised the study. PS, LGA, ABO, and EB designed the study. PS, LGA, ABO, EB conducted the stakeholder workshops. HMCK and LH contributed expertise through participation in workshops. PS and LGA accessed and verified data and developed the model with support from EB. PS drafted the manuscript and LGA, EB, HMCK, LH, JJM, CM, and ABO reviewed and edited the manuscript. All authors read and approved the final manuscript.

## Data sharing statement

The model used in this paper is freely available to download from the Open Science Foundation (OSF) depository following this link: https://osf.io/y6xnm/.

## Declaration of interests

The authors declare no conflict of interest.

## Appendix A Supplementary data

Supplementary Material

Abstract in Spanish
