# Supplementary material for: Actions against the double burden of malnutrition in Peru: a community-informed system dynamics model
Source: Lancet Reg Health Am. 2025 Apr 21;46:101102. doi: 10.1016/j.lana.2025.101102 (PMC12052979; doi:10.1016/j.lana.2025.101102)
Supplement: Supplementary Material [file mmc1.pdf]

# Supplementary Material

# Actions against the double burden of malnutrition in Peru: a community-informed system dynamics model

Paraskevi Seferidi[1], Laura Guzman-Abello[2], Ellis Ballard[3], Hilary M Creed-Kanashiro[4], Luis Huicho[5], J Jaime Miranda[6,7], Christopher Millett[1,8], Antonio Bernabe-Ortiz[6]

[1]Public Health Policy Evaluation Unit, School of Public Health, Imperial College London, London, UK

[2]Department of Design, School of Architecture and Design, Universidad de los Andes, Bogota, Colombia

[3]Social System Design Lab, Brown School at Washington University in St. Louis, St. Louis, USA

[4]Instituto de Investigación Nutricional, Lima, Peru

[5]Centro de Investigación en Salud Materna e Infantil, Centro de Investigación para el Desarrollo Integral y Sostenible and Facultad de Medicina, Universidad Peruana Cayetano Heredia, Lima, Peru

[6]CRONICAS Centre of Excellence in Chronic Diseases, Universidad Peruana Cayetano Heredia, Lima, Peru

[7]Sydney School of Public Health, Faculty of Medicine and Health, University of Sydney, Sydney, Australia

[8]NOVA National School of Public Health, Public Health Research Centre, Comprehensive Health Research Center, NOVA University Lisbon, Lisbon, Portugal

**Table of Contents**

Table of Contents ........ 2

Supplementary Text S1. System dynamics ........ 3

Supplementary Text S2. Behaviour reproduction ........ 4

Supplementary Text S3. Integration error ........ 6

Supplementary Text S4. Sensitivity Analyses ........ 12

Supplementary Text S5. Extreme conditions ........ 24

Supplementary Table S1. Table with variables used in the model. The variable names, units of measure, value (for constants) and equations (for all other variables), brief description, and source, where relevant, are presented. The variables are presented by type of variable which include: Constants, Auxiliaries, Stocks, Flow, and Smooth variables ........ 32

Supplementary Table S2. Main feedback mechanisms that drive model behaviour ........ 51

Supplementary Table S3. Modelling assumptions made under each scenario ........ 52

Supplementary Figure S1. Behaviour over-time graph for ultra-processed food consumption, under different scenarios ........ 53

References ........ 54

**Supplementary Text S1. System dynamics**

System Dynamics is a methodology used to develop computer simulation models that are built based on the way in which the elements of a system interact to inform the system's dynamic complexity, the sources of resistance to policies that change the system and the design of more effective policies. It is based on the theory of nonlinear dynamics, decision making and feedback[1]. System dynamics models help understand the behaviour of a system based on the way it is organized; solve problems; expose new discussions, questions and limitations; expose uncertainties; test implicit mental models of decision makers; promote learning; and support decision-making processes[1–4].

System Dynamics models diagrammatically describe the structure and interrelationships of a system and represent the operations of a system mainly using two tools: Causal Loop Diagrams (CLD) and Stock and Flow Diagrams. Causal Loop Diagrams represent the feedback structure in a system. Feedback is a process in which decision making affects the system and in turn that system produces information for making new decisions. CLDs consist of variables connected by arrows that denote that there is a causal relationship between the variables. Each causal relationship (arrow) has a polarity that denotes the direction of the change: positive polarity indicates that a change in the cause produces a change in the same direction in the effect and negative polarity indicates that a change in the cause produces a change in the opposite direction on the effect [1]. When the effect of one variable over the other takes considerable time (is delayed) arrows are marked with two perpendicular lines. A combination of multiple variables connected through causal relationships eventually conform feedback loops. Systems have two types of feedback loops: self-balancing that oppose change in a system and are associated with processes that seek balance (in models represented with a letter B or a negative sign); and self-reinforcing that amplify change in the system and are associated with processes that generate increasing change (in models represented with a letter R or a positive sign) [1].

Stock and Flow Diagrams emphasise the operational structure that underlies the system, that is, how it works. They are constituted by stocks, flows, auxiliary variables, causal relationships, delays and decision rules (equations) (Sterman [1]. Stocks are variables that represent accumulations over time, which occur when the decrease process has a lower rate than the increase process. Stocks characterize the state of the system and generate the information on which decisions and actions are based. They also create delays by accumulating the difference between the increase and decrease processes. Flows are processes that add or subtract material to stocks and are the main reason for their increase or decrease. Auxiliary variables are intermediate variables that may be a function of the information provided by the levels, flows or other auxiliary variables; or they may also contain exogenous and constant information. Decision rules are mathematical equations that operationalize decision making processes of actors in the system and are necessary for model simulation.

## Supplementary Text S2. Behaviour reproduction

This test assesses if the model reproduces the behaviour of interest in the system, if it endogenously generates the problem being assessed, if it generates various modes of behaviour observed in the real system and if the frequencies and phase relationships among the variables match the data[5]. In this case we will compare simulation results with observed data between 2000 and 2020 for child (0-15 years) and adult (15-49 years) population, obtained from Instituto Nacional de Estadística e Informática (INEI) INEI[6], Peru, as well as prevalence of under-5 stunting and adult overweight in Peru, obtained from repeated surveys from the Encuesta Demográfica y de Salud Familiar (ENDES) in Peru, 2000-2019[7]. We were not able to compare our results with overweight or stunting prevalence in children 5-15 years old, as no such data were available at national level in Peru.

| Variable | Results | Observations |
|---|---|---|
| Total adult population | 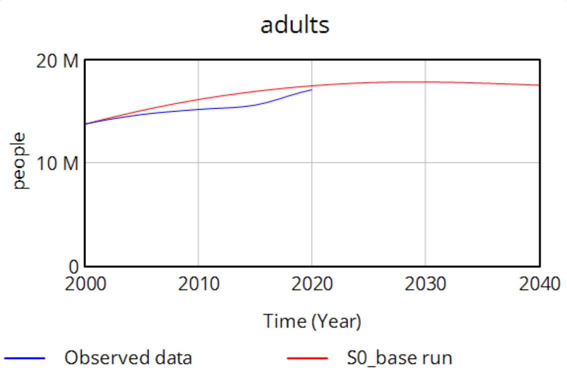 | Overall, the simulation produces similar patterns of behaviour for the total adult population compared to the observed data. The observed data show a slower increase between approximately 2005 and 2015, which then increases more rapidly between 2015 and 2020. In contrast, the simulation produces a smoother increase which, however, catches up with observed data by 2020. This is likely to be because we only used estimates of change in mortality and fertility over 5-year periods, which provide enough accuracy for the purposes of this model. |
| Total child population | 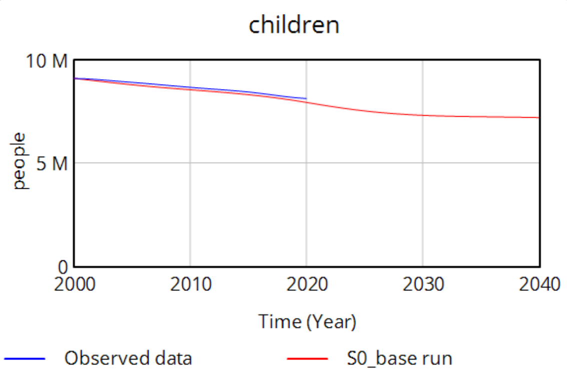 | The simulation produces very similar patterns of behaviour for the total child population compared to observed data. There is almost no behavioural or numerical deviation observed. |

| Variable | Results | Observations |
|---|---|---|
| Adult overweight prevalence | 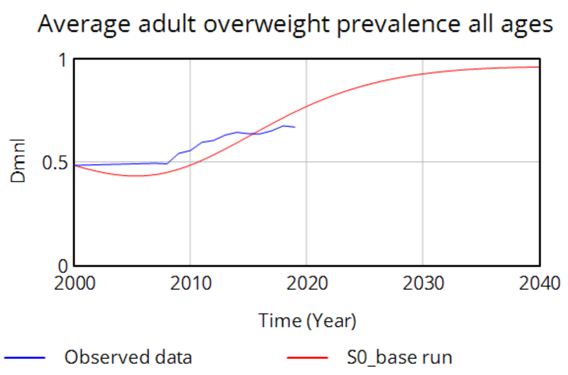 | Overall, the simulation produces similar patterns of behaviour for the average adult overweight prevalence compared to the observed data. There are no observed data on overweight prevalence between 2000 and 2005, which makes it harder to validate the slight deep that the model produces during these years. However, the simulation generally replicates the over-time behaviour of adult overweight prevalence after 2005, which shows a more rapid increase initially, which then gradually plateaus. There are some numerical differences between the simulated and observed data, however, we do not think that this is likely to impact the conclusions of this study. |
| Under-5 stunting | 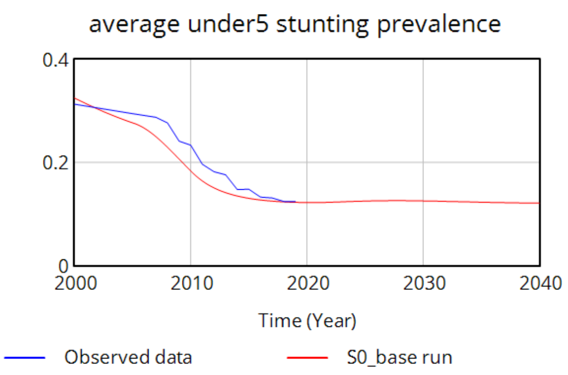 | Overall, the simulation produces similar patterns of behaviour for the average under-5 stunting prevalence compared to the observed data. Similarly to overweight, there are no observed data on stunting prevalence between 2000 and 2005, which makes it harder to validate the behaviour that the model produces during these years. However, the simulation generally replicates the over-time behaviour of under-stunting prevalence in Peru, which shows slight increase between 2000 and 2008, which then becomes more rapid until approximately 2016, when it plateaus. There are some numerical differences between the simulated and observed data, however, we do not think that this is likely to impact the conclusions of this study. |

## Supplementary Text S3. Integration error

System Dynamics models are systems of simultaneous differential equations that are solved algorithmically by numerical methods such as the Euler or Runge-Kutta fourth-order method. Additionally, when running these models, the size of the interval in which the corresponding calculations are made must be defined, that is, the size of the time step (also known as DT). This test assesses whether the results produced by the model are sensitive to changes in the integration method and in the DT value[5].

1. Euler method

We first ran the model using Euler´s method changing the values of the DT from 1 to 0.0078127 (in total 8 simulations).

<table>
<tr><th>Description</th><th>Simulation results</th><th>Observations</th></tr>
<tr>
<td>Simulation of Average adult overweight prevalence with Euler method changing DT from 1 to 0.0078125 (8 simulations).<br><br>% Difference is calculated for the simulated values at the midpoint of the simulation (t = 2020) and at the end of the simulation (t = 2040) compared to the smallest DT.</td>
<td>

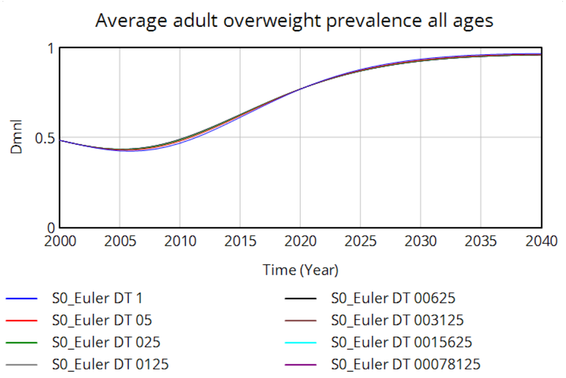


<table>
<tr><th colspan="3">Average adult overweight prevalence all ages</th></tr>
<tr><th>Integration type; Time step (DT)</th><th>% Difference (compared to smallest DT value)<br><br>t = 2020</th><th>% Difference (compared to smallest DT value)<br><br>t = 2040</th></tr>
<tr><td>Euler; DT 0.0078125</td><td>0.000%</td><td>0.000%</td></tr>
<tr><td>Euler; DT 0.015625</td><td>0.000%</td><td>0.008%</td></tr>
<tr><td>Euler; DT 0.03125</td><td>-0.001%</td><td>0.018%</td></tr>
<tr><td>Euler; DT 0.0625</td><td>-0.002%</td><td>0.050%</td></tr>
<tr><td>Euler; DT 0.125</td><td>-0.006%</td><td>0.103%</td></tr>
<tr><td>Euler; DT 0.25</td><td>-0.017%</td><td>0.184%</td></tr>
<tr><td>Euler; DT 0.5</td><td>-0.039%</td><td>0.391%</td></tr>
</table>

</td>
<td>The S-shape of the curve is slightly more pronounced as DT increases.<br><br>For all DT values the % difference is less than 1% both at the midpoint of the simulation (t = 2020) and at the end of the simulation (t = 2040).</td>
</tr>
</table>

<table>
<tr><th>Description</th><th>Simulation results</th><th>Observations</th></tr>
<tr><td></td><td><table><tr><td>Euler; DT 1</td><td>-0.104%</td><td>0.791%</td></tr></table></td><td></td></tr>
<tr><td>Simulation of Average under-5 stunting prevalence with Euler method changing DT from 1 to 0.0078125 (8 simulations).<br><br>% Difference is calculated for the simulated values at the midpoint of the simulation (t = 2020) and at the end of the simulation (t = 2040) compared to the smallest DT</td><td>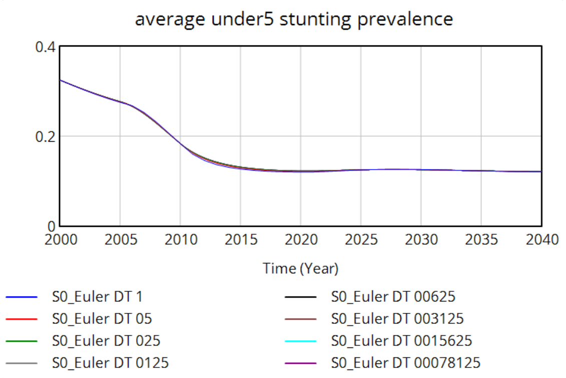

<table>
<tr><th colspan="3">Average adult overweight prevalence all ages</th></tr>
<tr><th>Integration type; Time step (DT)</th><th>% Difference (compared to smallest DT value)<br><br>t = 2020</th><th>% Difference (compared to smallest DT value)<br><br>t = 2040</th></tr>
<tr><td>Euler; DT 0.0078125</td><td>0.000%</td><td>0.000%</td></tr>
<tr><td>Euler; DT 0.015625</td><td>-0.020%</td><td>-0.006%</td></tr>
<tr><td>Euler; DT 0.03125</td><td>-0.046%</td><td>-0.012%</td></tr>
<tr><td>Euler; DT 0.0625</td><td>-0.127%</td><td>-0.033%</td></tr>
<tr><td>Euler; DT 0.125</td><td>-0.261%</td><td>-0.067%</td></tr>
<tr><td>Euler; DT 0.25</td><td>-0.473%</td><td>-0.116%</td></tr>
<tr><td>Euler; DT 0.5</td><td>-1.009%</td><td>-0.266%</td></tr>
<tr><td>Euler; DT 1</td><td>-2.080%</td><td>-0.623%</td></tr>
</table></td><td>The deep of the curve is slightly lower as DT increases, especially for the largest DT = 1.<br><br>The % difference at the mid-point of the simulation (t = 2020) is more than 1% for DT = 0.5 and DT = 1, which indicates that it is advisable to use lower values of DT to correctly capture the dynamics of this variable.<br><br>For all the other DT values the % difference is less than 1% both at the midpoint of the simulation (t = 2020) and at the end of the simulation (t = 2040).</td></tr>
</table>

2. Runge-Kutta fourth order

We then ran the model using Runge-Kutta´s fourth-order method changing the values of the DT from 1 to 0.0078127 (in total 8 simulations).

<table>
<tr><th>Description</th><th>Simulation results</th><th>Observations</th></tr>
<tr>
<td>Simulation of Average adult overweight prevalence with Runge-Kutta´s fourth-order method changing DT from 1 to 0.0078125 (8 simulations).<br><br>% Difference is calculated for the simulated values at the midpoint of the simulation (t = 2020) and at the end of the simulation (t = 2040) compared to the smallest DT.</td>
<td>
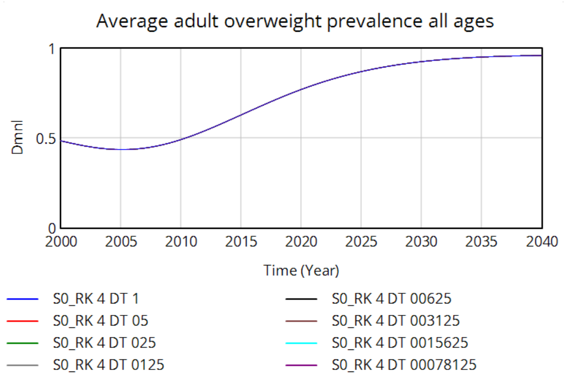

<table>
<tr><th colspan="3">Average adult overweight prevalence all ages</th></tr>
<tr><th>Integration type; Time step (DT)</th><th>% Difference (compared to smallest DT value)<br><br>t = 2020</th><th>% Difference (compared to smallest DT value)<br><br>t = 2040</th></tr>
<tr><td>Runge-Kutta 4; DT 0.0078125</td><td>0.000%</td><td>0.000%</td></tr>
<tr><td>Runge-Kutta 4; DT 0.015625</td><td>0.000%</td><td>0.000%</td></tr>
<tr><td>Runge-Kutta 4; DT 0.03125</td><td>0.000%</td><td>0.001%</td></tr>
<tr><td>Runge-Kutta 4; DT 0.0625</td><td>-0.001%</td><td>0.001%</td></tr>
<tr><td>Runge-Kutta 4; DT 0.125</td><td>-0.001%</td><td>0.001%</td></tr>
<tr><td>Runge-Kutta 4; DT 0.25</td><td>-0.002%</td><td>0.002%</td></tr>
<tr><td>Runge-Kutta 4; DT 0.5</td><td>-0.005%</td><td>0.004%</td></tr>
<tr><td>Runge-Kutta 4; DT 1</td><td>-0.009%</td><td>0.008%</td></tr>
</table>
</td>
<td>There is no change in behavioural patterns when the DT changes.<br><br>For all DT values the % difference is less than 0.01% both at the midpoint of the simulation (t = 2020) and at the end of the simulation (t = 2040).</td>
</tr>
</table>

<table>
<tr><th>Description</th><th>Simulation results</th><th>Observations</th></tr>
<tr>
<td>Simulation of Average under-5 stunting prevalence with Runge-Kutta´s fourth-order method changing DT from 1 to 0.0078125 (8 simulations).<br><br>% Difference is calculated for the simulated values at the midpoint of the simulation (t = 2020) and at the end of the simulation (t = 2040) compared to the smallest DT.</td>
<td>
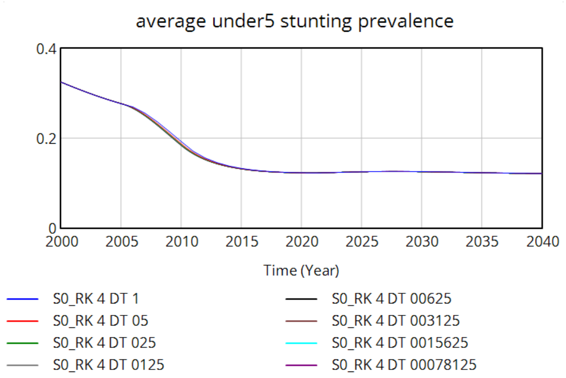

<table>
<tr><th colspan="3">Average adult overweight prevalence all ages</th></tr>
<tr><th>Integration type; Time step (DT)</th><th>% Difference (compared to smallest DT value)<br><br>t = 2020</th><th>% Difference (compared to smallest DT value)<br><br>t = 2040</th></tr>
<tr><td>Runge-Kutta 4; DT 0.0078125</td><td>0.000%</td><td>0.000%</td></tr>
<tr><td>Runge-Kutta 4; DT 0.015625</td><td>0.001%</td><td>0.000%</td></tr>
<tr><td>Runge-Kutta 4; DT 0.03125</td><td>0.001%</td><td>0.001%</td></tr>
<tr><td>Runge-Kutta 4; DT 0.0625</td><td>0.007%</td><td>0.003%</td></tr>
<tr><td>Runge-Kutta 4; DT 0.125</td><td>0.015%</td><td>0.008%</td></tr>
<tr><td>Runge-Kutta 4; DT 0.25</td><td>0.033%</td><td>0.016%</td></tr>
<tr><td>Runge-Kutta 4; DT 0.5</td><td>0.072%</td><td>0.034%</td></tr>
<tr><td>Runge-Kutta 4; DT 1</td><td>0.169%</td><td>0.071%</td></tr>
</table>
</td>
<td>The deep of the curve is slightly higher as DT increases, especially for the largest DT = 1.<br><br>For all DT values the % difference is less than 0.2% both at the midpoint of the simulation (t = 2020) and at the end of the simulation (t = 2040).</td>
</tr>
</table>

3. Comparison between Euler´s method and Runge-Kutta 4

We then compared the results obtained when using the same DT value but changing the integration method (Euler and Runge-Kutta 4). Results only show significant differences in the children 0-1 stock for DT values higher than 0.0625. This is consistent with the previous results.

<table>
<tr><th>Description</th><th>Simulation results</th><th>Observations</th></tr>
<tr>
<td>Simulation of Average adult overweight prevalence with both Euler’s and Runge-Kutta´s fourth-order methods, in order to compare the two methods across different DTs, from 1 to 0.0078125 (16 simulations).<br><br>% Difference is calculated for the simulated values at the midpoint of the simulation (t = 2020) and at the end of the simulation (t = 2040) for Euler method compared to the Runge-Kutta´s fourth-order method, across the 8 DT points.</td>
<td>
<table>
<tr><th colspan="3">Average adult overweight prevalence all ages</th></tr>
<tr><th>Time step (DT)</th><th>% Difference (Euler method compared to Runge-Kutta´s fourth-order method)<br><br>t = 2020</th><th>% Difference (Euler method compared to Runge-Kutta´s fourth-order method)<br><br>t = 2040</th></tr>
<tr><td>DT 0.0078125</td><td>0.000%</td><td>0.006%</td></tr>
<tr><td>DT 0.015625</td><td>-0.001%</td><td>0.014%</td></tr>
<tr><td>DT 0.03125</td><td>-0.002%</td><td>0.024%</td></tr>
<tr><td>DT 0.0625</td><td>-0.002%</td><td>0.056%</td></tr>
<tr><td>DT 0.125</td><td>-0.005%</td><td>0.108%</td></tr>
<tr><td>DT 0.25</td><td>-0.015%</td><td>0.189%</td></tr>
<tr><td>DT 0.5</td><td>-0.034%</td><td>0.393%</td></tr>
<tr><td>DT 1</td><td>-0.095%</td><td>0.789%</td></tr>
</table>
</td>
<td>For all DT values the % difference between the Euler method and the Runge-Kutta´s fourth-order method is less than 1% both at the midpoint of the simulation (t = 2020) and at the end of the simulation (t = 2040). However, the difference in larger, as DT increases, which further confirms the need to use a lower DT.</td>
</tr>
<tr>
<td>Simulation of Average under-5 stunting prevalence with both Euler’s and Runge-Kutta´s fourth-order methods, in order to compare the two methods across different DTs, from 1 to 0.0078125 (16 simulations).<br><br>% Difference is calculated for the simulated values at the midpoint of the simulation (t = 2020) and at the end of the simulation (t = 2040) for Euler method compared to the Runge-Kutta´s fourth-order method, across the 8 DT points.</td>
<td>
<table>
<tr><th colspan="3">Average adult overweight prevalence all ages</th></tr>
<tr><th>Time step (DT)</th><th>% Difference (Euler method compared to Runge-Kutta´s fourth-order method)<br><br>t = 2020</th><th>% Difference (Euler method compared to Runge-Kutta´s fourth-order method)<br><br>t = 2040</th></tr>
<tr><td>DT 0.0078125</td><td>-0.017%</td><td>-0.004%</td></tr>
<tr><td>DT 0.015625</td><td>-0.038%</td><td>-0.010%</td></tr>
<tr><td>DT 0.03125</td><td>-0.064%</td><td>-0.016%</td></tr>
<tr><td>DT 0.0625</td><td>-0.151%</td><td>-0.040%</td></tr>
<tr><td>DT 0.125</td><td>-0.293%</td><td>-0.079%</td></tr>
<tr><td>DT 0.25</td><td>-0.523%</td><td>-0.137%</td></tr>
<tr><td>DT 0.5</td><td>-1.096%</td><td>-0.304%</td></tr>
<tr><td>DT 1</td><td>-2.261%</td><td>-0.697%</td></tr>
</table>
</td>
<td>The % difference at the mid-point of the simulation (t = 2020) is more than 1% for DT = 0.5 and DT = 1, which indicates that it is advisable to use lower values of DT to correctly capture the dynamics of this variable.<br><br>For all the other DT values the % difference is less than 1% both at the midpoint of the simulation (t = 2020) and at the end of the simulation (t = 2040). However, the difference in larger, as DT increases, which further confirms the need to use a lower DT.</td>
</tr>
</table>

4. Conclusions
    a. There is no effect on behavioural patterns across varying DT values.
    b. Simulations show some numerical differences across varying DT values, which are higher than 1% for DT = 1 and DT = 0.5 at the mid-point of the simulation for the Average under-5 stunting variable. Thus, we chose DT = 0.25.
    c. Sterman[5] suggests that if there is no significant change between Euler or a higher order integration method (in this case Runge-Kutta 4), the Euler method should be used. Given that for the chosen DT = 0.25, the two methods show no significant differences, we chose the Euler´s integration method.

**Supplementary Text S4. Sensitivity Analyses**

This test assesses whether varying the assumptions of the model changes the results and conclusions of this study. We tested numerical sensitivity, behavioural sensitivity, and policy sensitivity. We used the Sensitivity2All tool in Vensim to explore the impact of changing all constant parameters of the model by +/-10% on two endogenous variables in the model: Average adult overweight prevalence (mean of all age groups) and Average under-5 stunting prevalence. First, we explored numerical sensitivity by estimating the percentage change in adult overweight and under-5 stunting integrated across the simulation period, under varying values of constant variables (Figure S3.1). Second, we explored behavioural sensitivity by observing the behaviour over time of adult overweight and under-5 stunting, under varying values of constant variables (Figure S3.2). Finally, we explored policy sensitivity by examining if numerical and behavioural sensitivity of the model varies under scenario 6 (Figure S3.3 and S3.4). Here, we only present policy sensitivity under scenario 6, as it includes all other scenarios.

Figure 1. Tornado graphs showing constant variables, their varying values, and their impact on (A) adult overweight and (B) under-5 stunting, expressed in percentage change across the simulation period. Only constant variables that accrue at least 1% change are presented in this figure.

(A) Average adult overweight prevalence

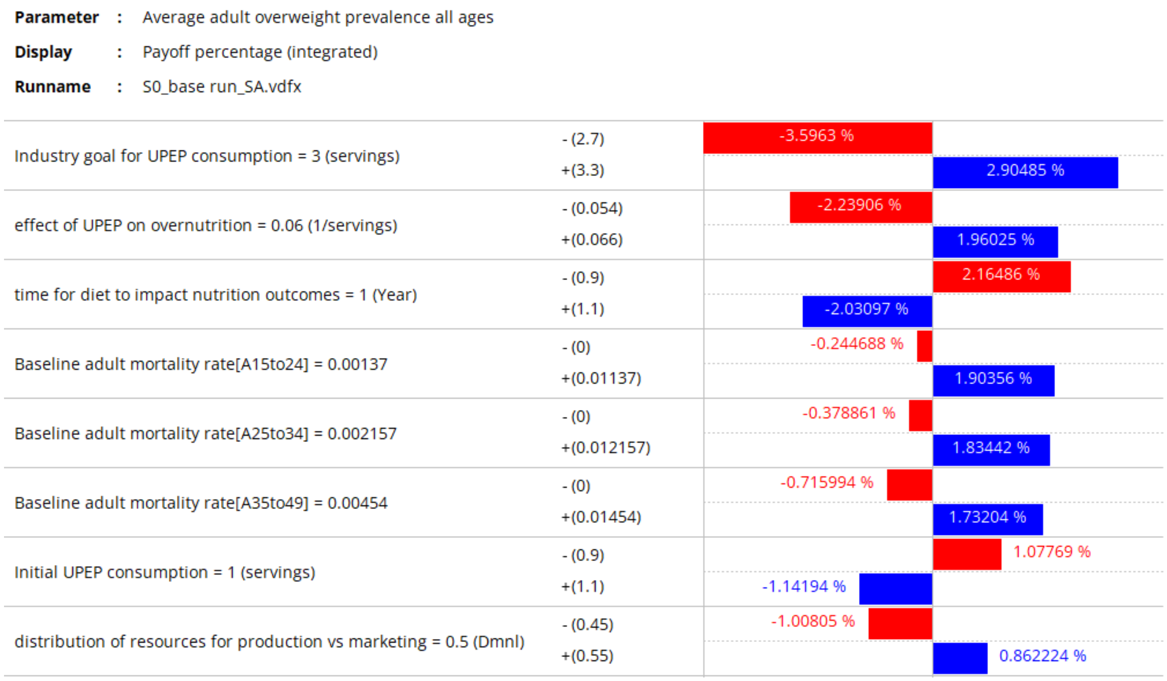

(B) Average under-5 stunting prevalence

**Parameter** : average under5 stunting prevalence
**Display** : Payoff percentage (integrated)
**Runname** : S0_base run_SA.vdfx

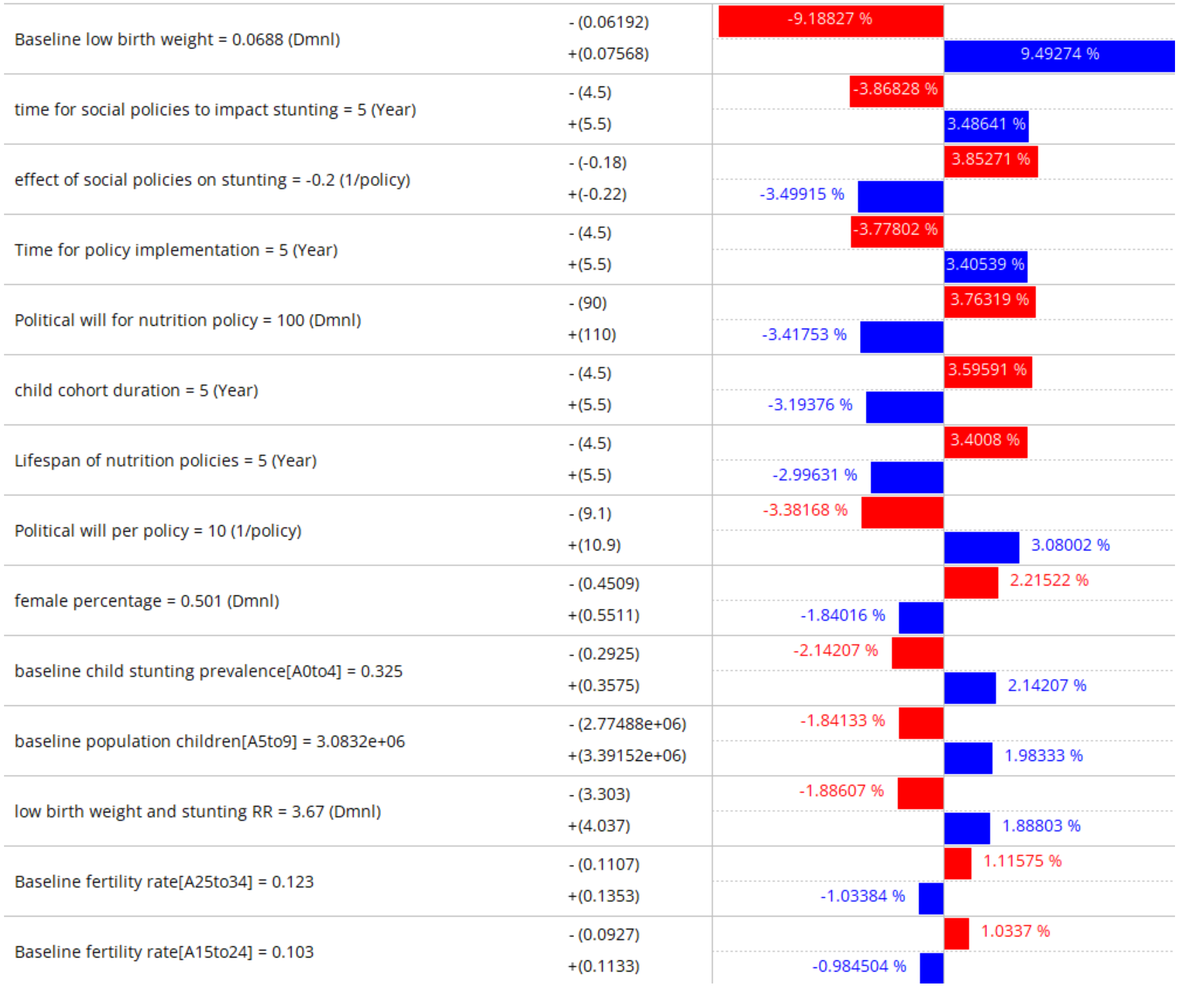

Figure 2. Behaviour over time graphs of (A) average adult overweight prevalence and (B) average under-5 stunting prevalence for varying constant variables by -10% (red line) or +10% (blue line) compared to baseline (black line). Graphs only for constant variables that accrue at least 1% change are presented.

(A) Average adult overweight prevalence

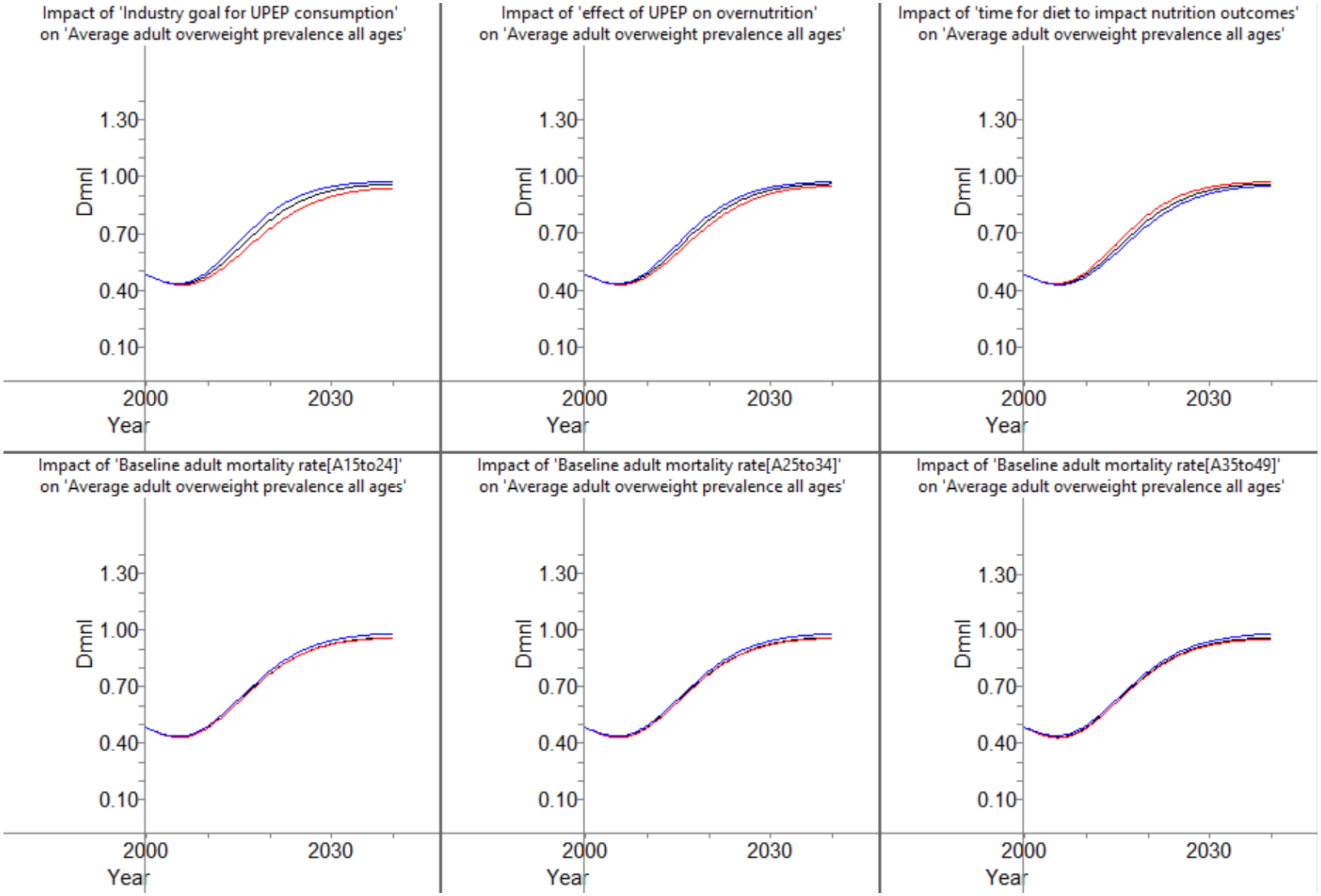

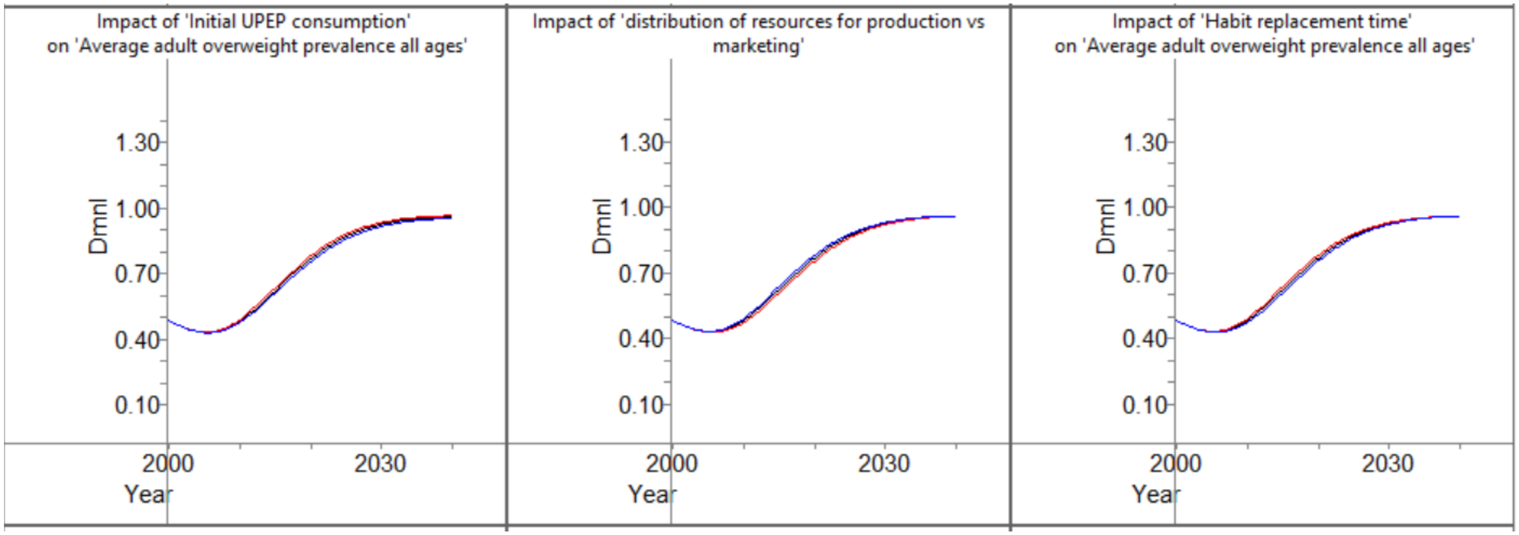


(B) Average under-5 stunting prevalence

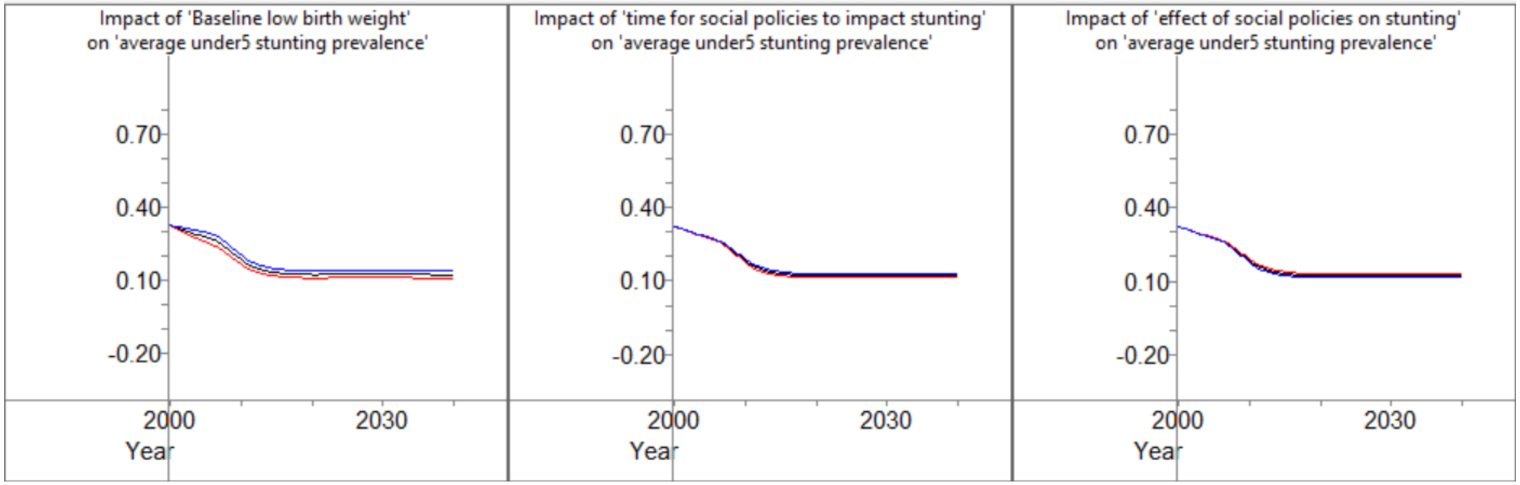

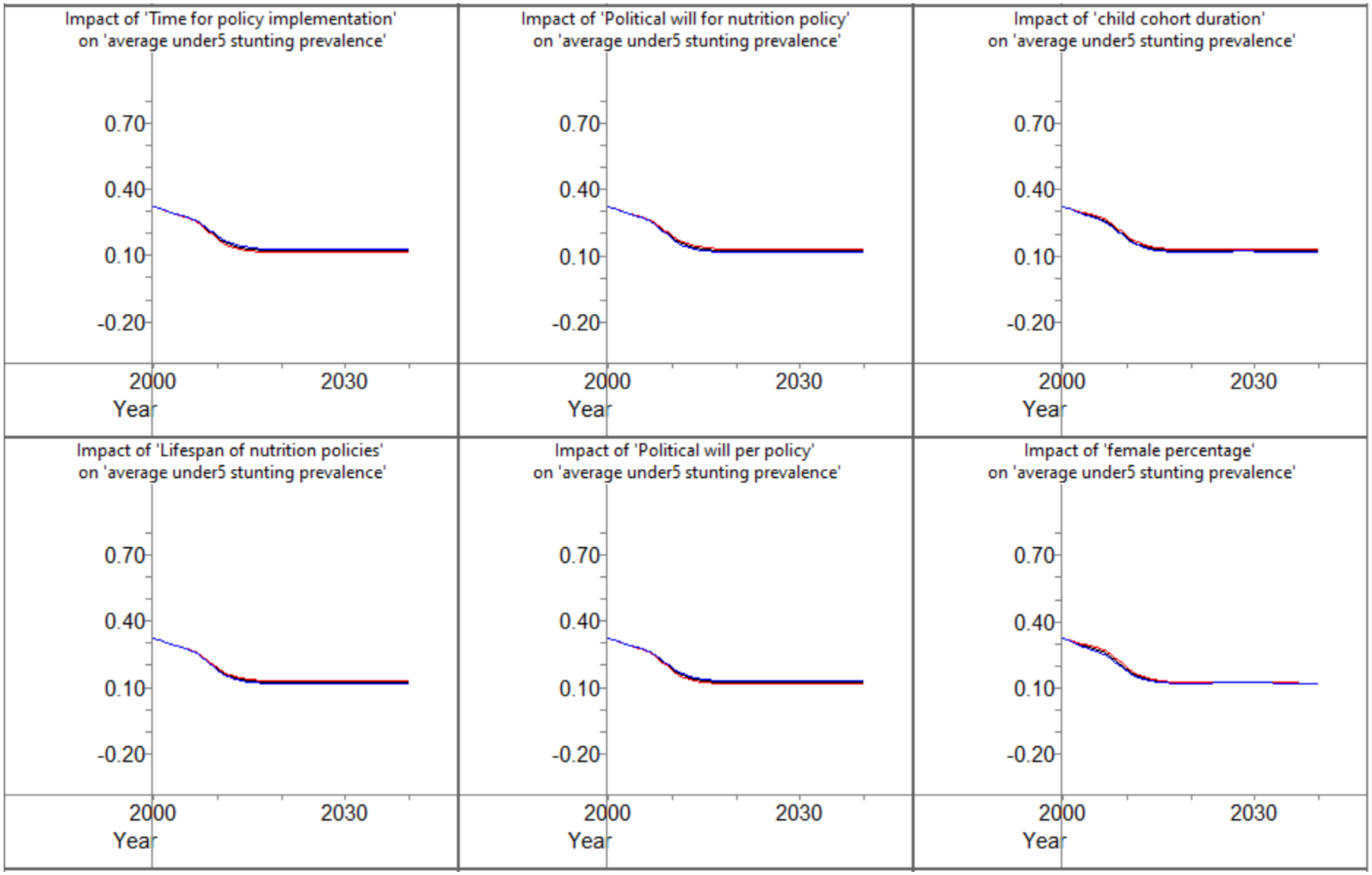
Impact of 'Time for policy implementation' on 'average under5 stunting prevalence'
Impact of 'Political will for nutrition policy' on 'average under5 stunting prevalence'
Impact of 'child cohort duration' on 'average under5 stunting prevalence'
Impact of 'Lifespan of nutrition policies' on 'average under5 stunting prevalence'
Impact of 'Political will per policy' on 'average under5 stunting prevalence'
Impact of 'female percentage' on 'average under5 stunting prevalence'
0.70
0.40
0.10
-0.20
2000
2030
Year

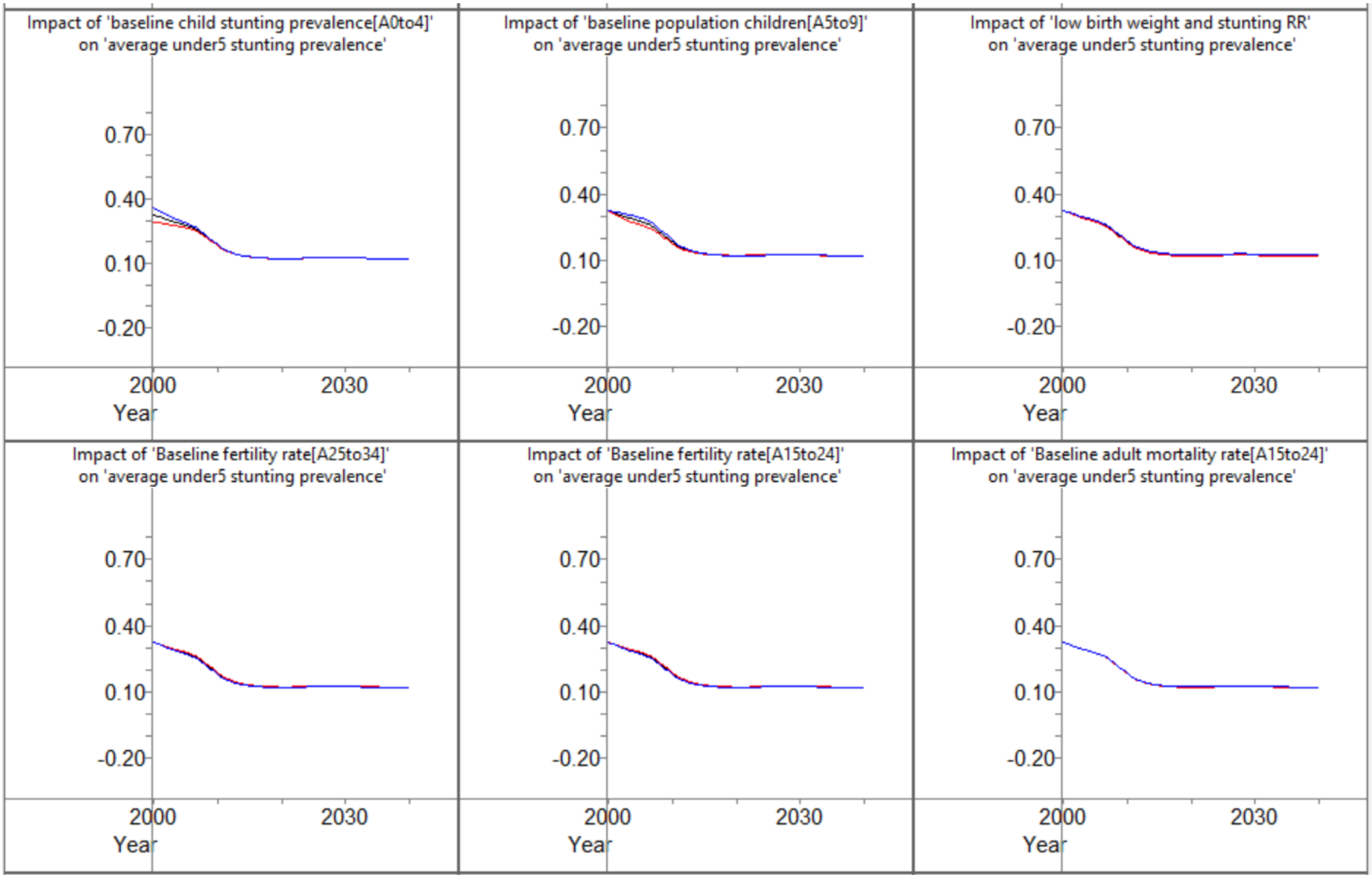

Impact of 'baseline child stunting prevalence[A0to4]' on 'average under5 stunting prevalence'
Impact of 'baseline population children[A5to9]' on 'average under5 stunting prevalence'
Impact of 'low birth weight and stunting RR' on 'average under5 stunting prevalence'
Impact of 'Baseline fertility rate[A25to34]' on 'average under5 stunting prevalence'
Impact of 'Baseline fertility rate[A15to24]' on 'average under5 stunting prevalence'
Impact of 'Baseline adult mortality rate[A15to24]' on 'average under5 stunting prevalence'
0.70
0.40
0.10
-0.20
2000
2030
Year

Figure 3. Tornado graphs showing constant variables, their varying values, and their impact on (A) adult overweight and (B) under-5 stunting, expressed in percentage change across the simulation period, under Scenario 6. Only constant variables that accrue at least 1% change are presented in this figure.

(A) Average adult overweight prevalence

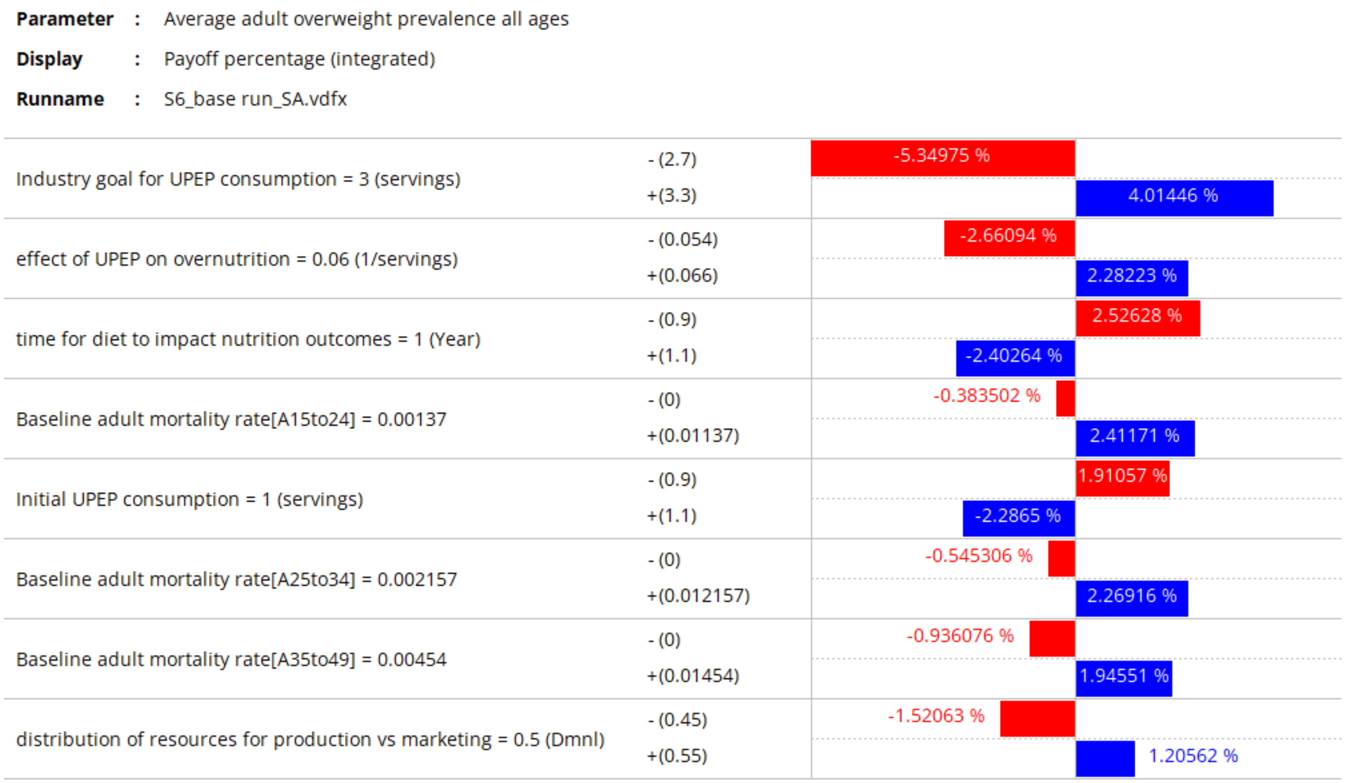

(B) Average under-5 stunting prevalence

**Parameter** : average under5 stunting prevalence

**Display** : Payoff percentage (integrated)

**Runname** : S6_base run_SA.vdfx

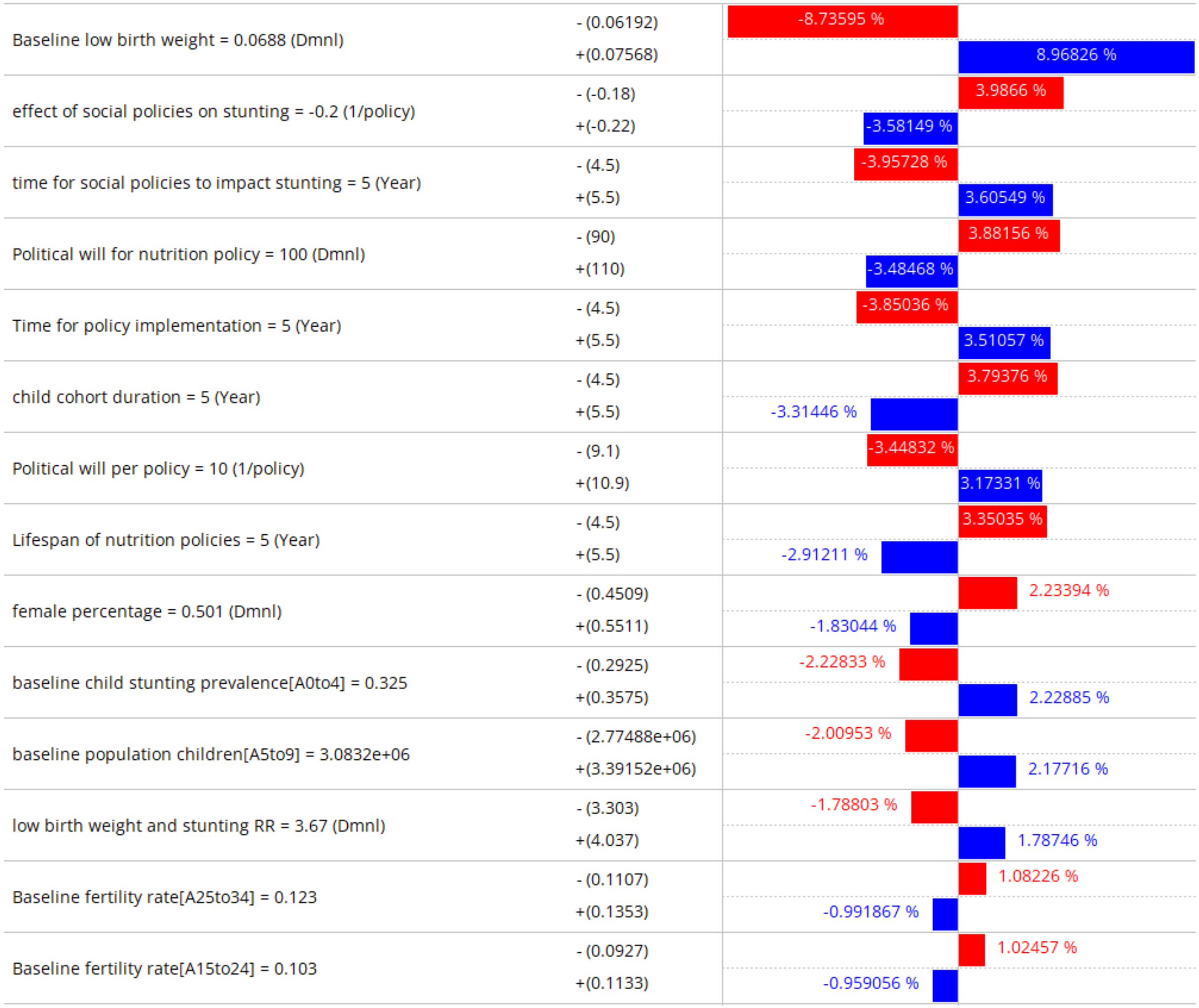

Figure 4. Behaviour over time graphs of (A) average adult overweight prevalence and (B) average under-5 stunting prevalence for varying constant variables by -10% (red line) or +10% (blue line) compared to baseline (black line), under Scenario 6. Graphs only for constant variables that accrue at least 1% change or indicate some behaviour sensitivity are presented.

(A) Average adult overweight prevalence

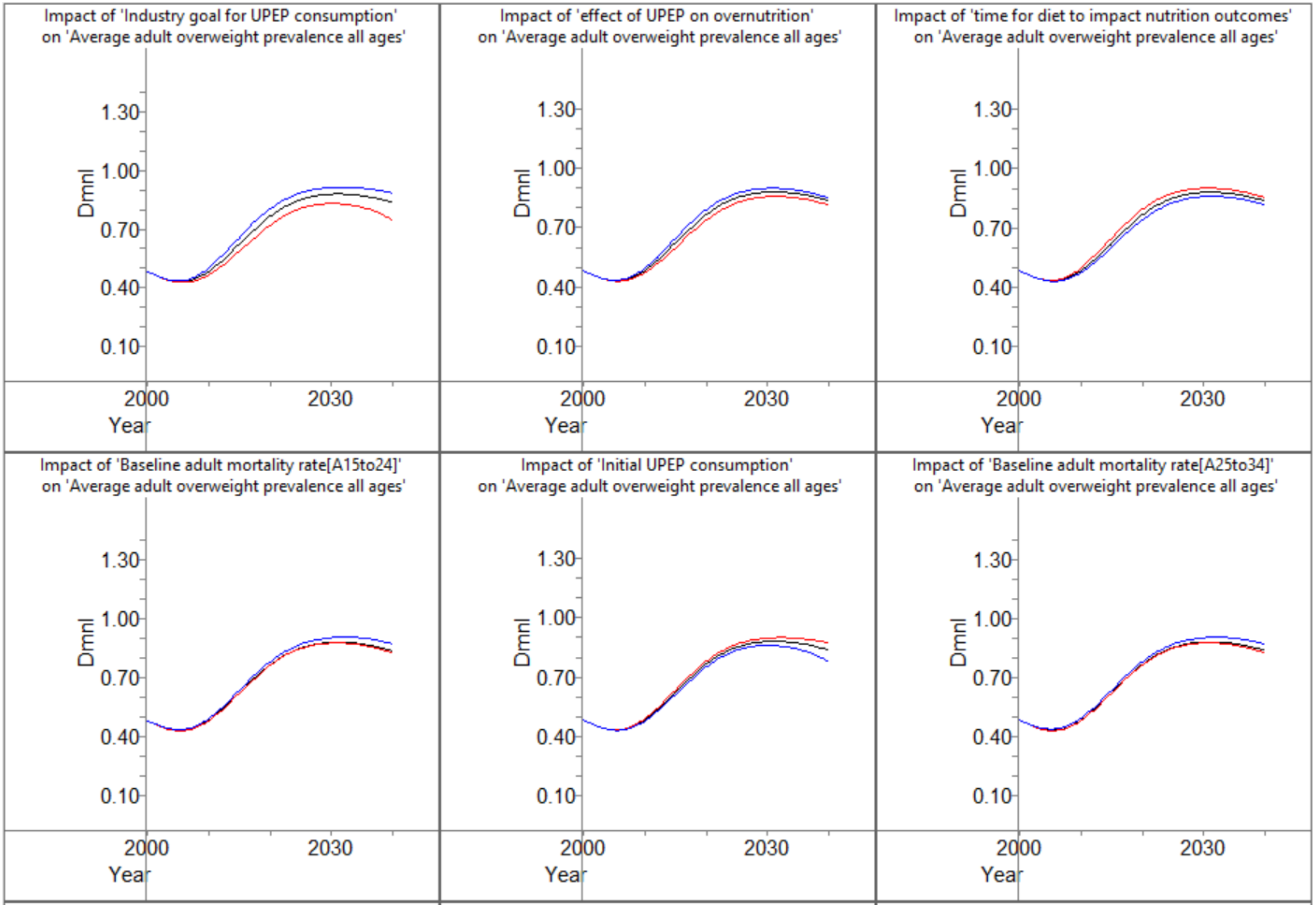

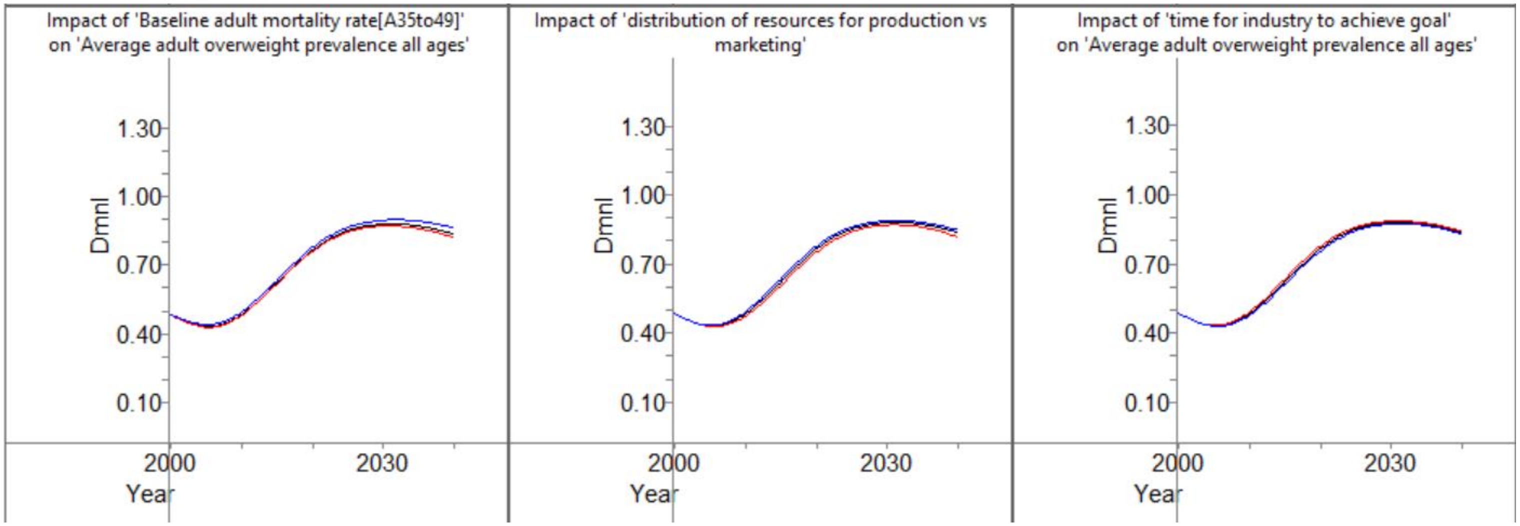


(B) Average under-5 stunting prevalence

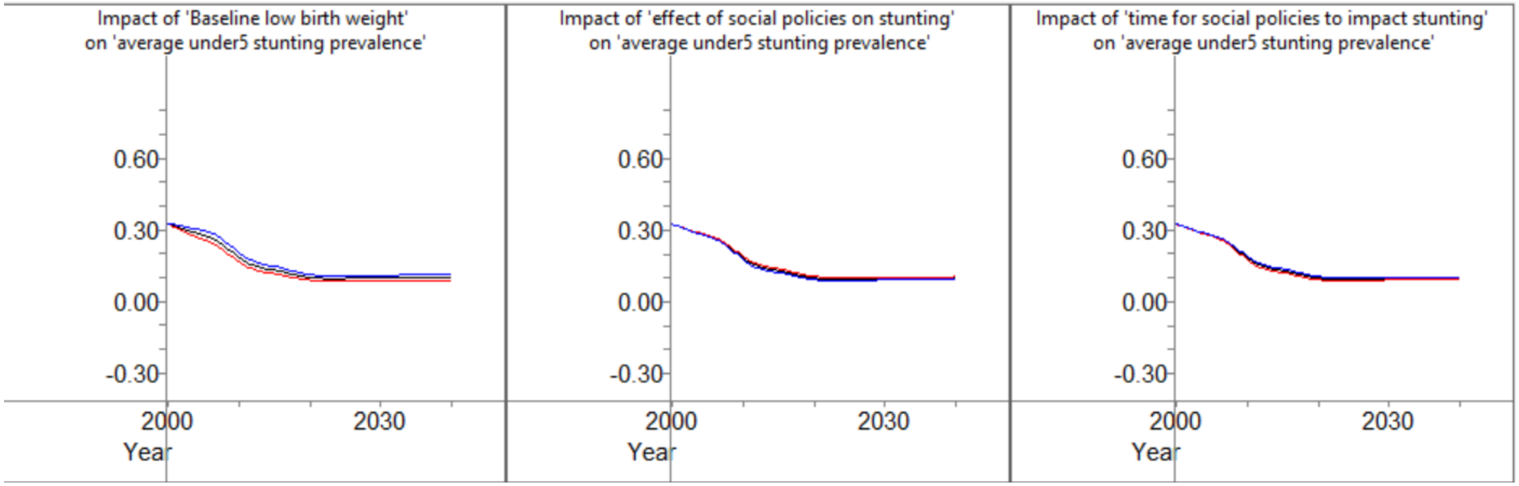

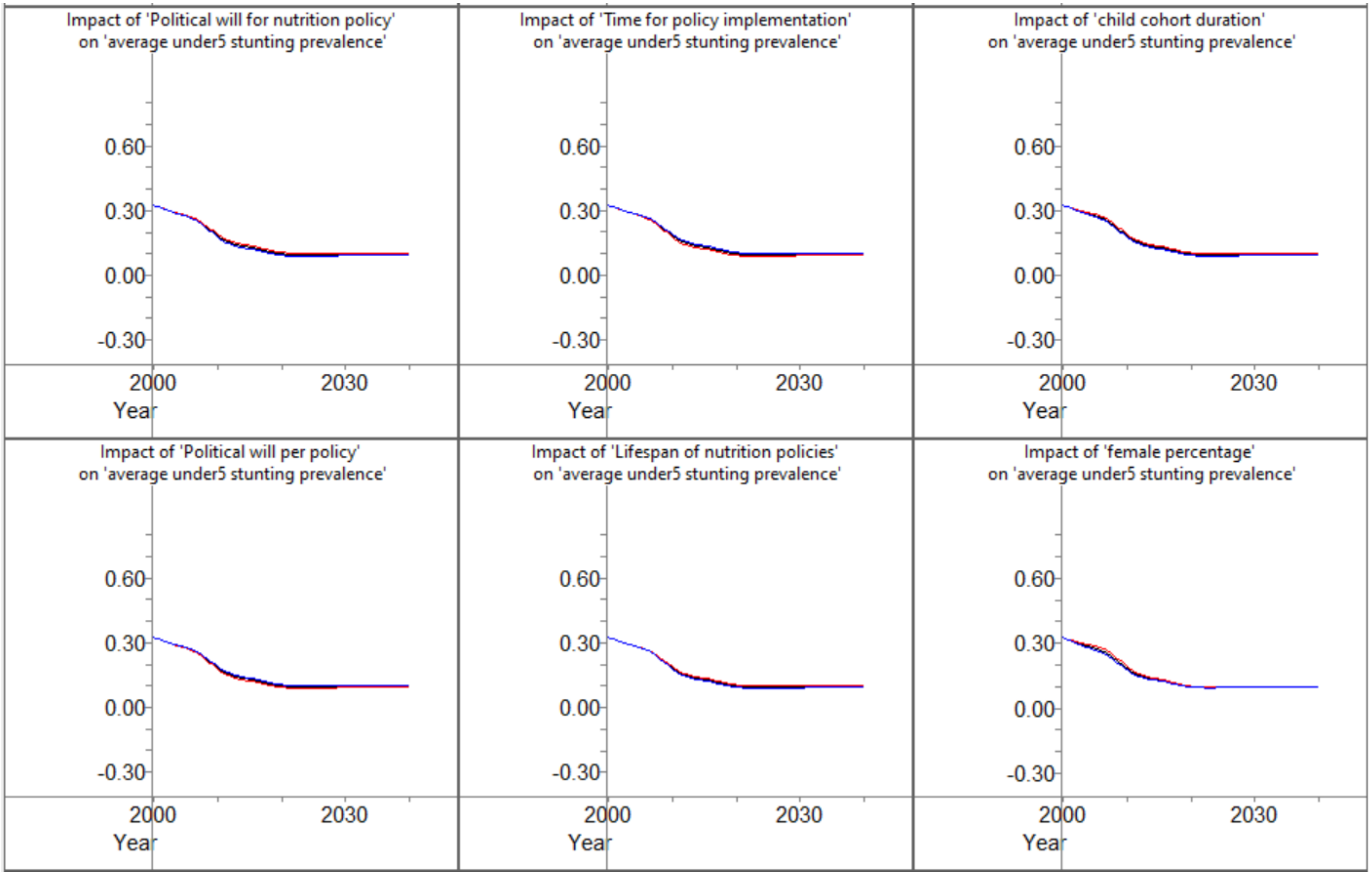


Conclusions

- Both adult overweight prevalence and under-5 stunting prevalence have relatively low numerical sensitivity for most constant variables in the model, with under-5 stunting prevalence displaying sensitivity to more constant variables (Number of variables that result in more than 1% change compared to baseline run: 15) compared to adult overweight prevalence (Number of variables that result in more than 1% change compared to baseline run: 9). For overweight, highest numerical sensitivity occurs for the variable “Industry goal for UPEP consumption”, which is not based on specific data but it is informed by UPEP consumption in other countries in the region (see Supplementary Table S1). Future model refinement can incorporate better understanding of UPEP industry operations in Peru. For under-5 stunting, highest numerical sensitivity occurs for the variable “Baseline low birth weight”, which is informed by national

birth registry data adjusted for trend (see Supplementary Table S1). This sensitivity is expected given that birth weight is a predictor of early life stunting. Under-5 stunting is also sensitive to variables that are informed by assumptions, e.g. time for social policies to impact stunting, effect of social policies on stunting, and political will for nutrition policy. This is supported by literature and workshop participants, who argued that reduction in stunting in Peru has been mainly the result of concerted political efforts to incorporate social policies, such as cash assistance programmes, in nutrition policy. However, future model refinement can further explore the mechanisms through which such policies occurred and impacted stunting in Peru. Overall, numerical sensitivity of adult overweight and under-5 stunting is minimal for most variables and does not change the conclusions of our study.

- Behavioural sensitivity does not occur for adult overweight for any of the variables. Under-5 stunting shows some minimal behavioural sensitivity to changes in baseline low birth weight, baseline under-5 stunting prevalence, and baseline population children [5-9]. Changes in these variables show a slightly different rate of change in the first 5 years of the simulation compared to the base run. However, these estimates are derived from high quality data and are not based on assumptions, reducing uncertainty about their impacts.

- Policy analysis shows that the model does not ensue any notable sensitivity under different scenarios. There is some behaviour sensitivity for adult overweight prevalence under Scenario 6 for variables that impact the association between overweight and UPEP consumption, such as Industry goal for UPEP consumption and Initial UPEP consumption. Sensitivity analyses for these variables show that overweight will reduce even more rapidly compared to the baseline run for Scenario 6. This is expected as the simulated reduction of adult overweight under Scenario 6 occurs through the impact of UPEP. So, any changes that enhance this impact are likely to have an even more significant effect on overweight.

**Supplementary Text S5. Extreme conditions**

This test assesses whether the equations and results of the model make sense when subjected to extreme values, parameters, and policies. We examined extreme conditions for variables that are based on significant assumptions, as identified in the parameter assessment test and to which adult overweight and under-5 stunting variables are most sensitive, according to the Sensitivity Analysis (SA).

<table>
<tr><th>Variable</th><th>Description</th><th>Simulation results</th><th>Simulations as expected</th></tr>
<tr><td>Industry goal for UPEP consumption ( = 3 servings)</td><td>“Industry goal for UPEP” is based on an assumption and was estimated based on UPEP sales in other countries in Latin America. SA also showed that average adult overweight prevalence is numerically sensitive to changes in this variable. We varied the “Industry goal for UPEP” to take values of min = 1 and max = 6. Under the minimum extreme scenario, we expect that overweight will not increase, given that the industry goal for UPEP will be equal to the actual UPEP consumption, and thus it will make no efforts to further increase UPEP. Under the maximum extreme scenario, we expect that overweight prevalence will increase more rapidly compared to the base run, as industry efforts to increase UPEP consumption will be more intense. We do not expect under-5 stunting prevalence to show a notable change, as UPEP consumption has no direct effect on stunting.</td><td>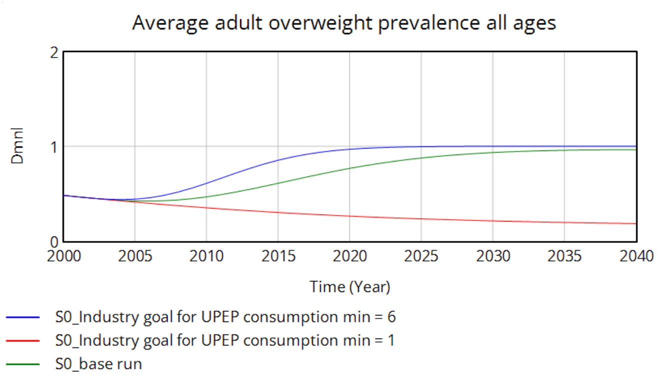

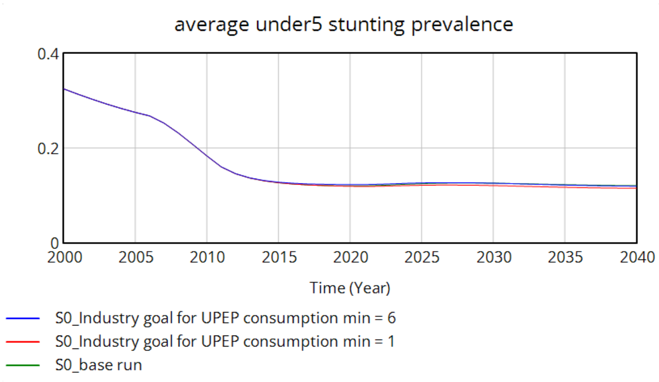
</td><td>Yes</td></tr>
</table>

| Variable | Description | Simulation results | Simulations as expected |
|---|---|---|---|
| Effect of UPEP on overnutrition (= 0.06 1/servings) | “Effect of UPEP on overnutrition” is estimated from a Relative Risk (RR) = 1.18 for high vs low consumers of UPEP, obtained from a published meta-analysis, and adjusted to reflect change per serving of consumption. SA showed that adult overweight prevalence is numerically sensitive to changes in this variable. We varied the “Effect of UPEP on overnutrition” to take values of min = 0, i.e. UPEP intake does not impact overweight (RR = 1), and max = 1, i.e. each serving increase in UPEP doubles the risk of overweight. Under these extreme scenarios, we expect that overweight will not increase (for min) and will very rapidly increase (for max) compared to the base run. We do not expect under-5 stunting prevalence to show a notable change, as UPEP consumption has no direct effect on stunting. | 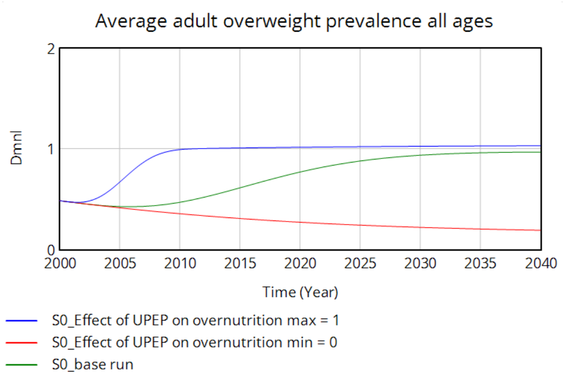<br>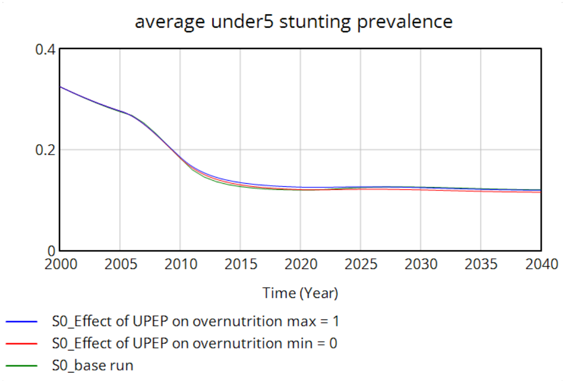 | Yes |

<table>
<tr><th>Variable</th><th>Description</th><th>Simulation results</th><th>Simulations as expected</th></tr>
<tr><td rowspan="2">time for diet to impact nutrition outcomes (=1 year)</td><td rowspan="2">“time for diet to impact nutrition outcomes” is based on an assumption, calibrated against observed overweight prevalence. SA showed that adult overweight prevalence is numerically sensitive to changes in this variable. We varied time for diet to impact nutrition outcomes to take values min = 1/12, i.e. it takes 1 month for change in population diet to change population overweight and stunting outcomes, and max = 5, i.e. it takes 5 years for change in population diet to change population overweight and stunting outcomes. Under these extreme scenarios, we expect that overweight will increase faster (for min) and slower (for max) compared to the base run. We do not expect under-5 stunting prevalence to show a notable change, because although we model an impact of MPF consumption on stunting, this impact is much smaller compared to the impact of social policies that mainly drive stunting reduction.</td><td>
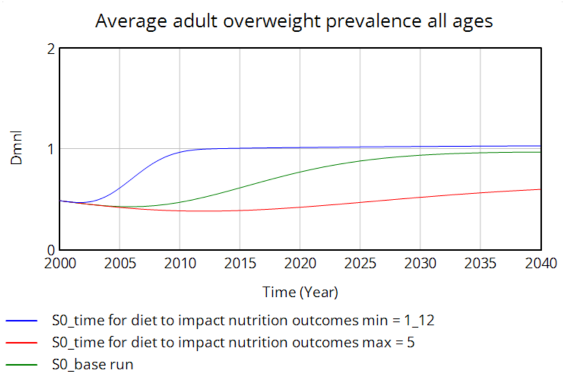

</td><td rowspan="2">Yes</td></tr>
<tr><td>
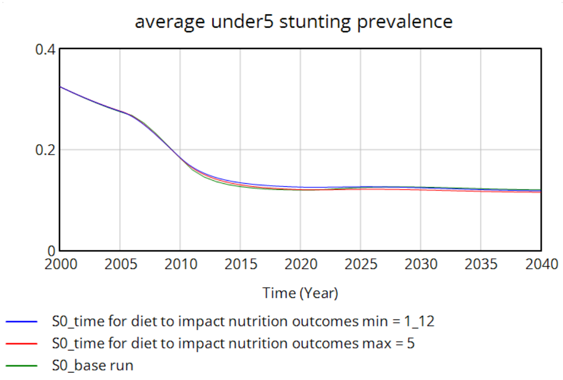

</td></tr>
</table>

<table>
<tr><th>Variable</th><th>Description</th><th>Simulation results</th><th>Simulations as expected</th></tr>
<tr><td>Time for social policies to impact stunting ( = 5 years)</td><td>“Time for social policies to impact stunting” is based on an assumption, calibrated against observed stunting prevalence. SA showed that under-5 stunting prevalence is numerically sensitive to changes in this variable. We varied “Time for social policies to impact stunting” to take values min = 1, i.e. it takes 1 year for social policies to change population stunting, and max = 10, i.e. it takes 10 years for social policies to change population stunting. Under these extreme scenarios, we expect that overweight will not change at all, as social policies do not impact overweight in this model. We expect under-5 stunting to decrease faster (for min) and slower (for max) compared to the base run, as it will take less and more time respectively for its main driver of decline (social policies) to work.</td><td>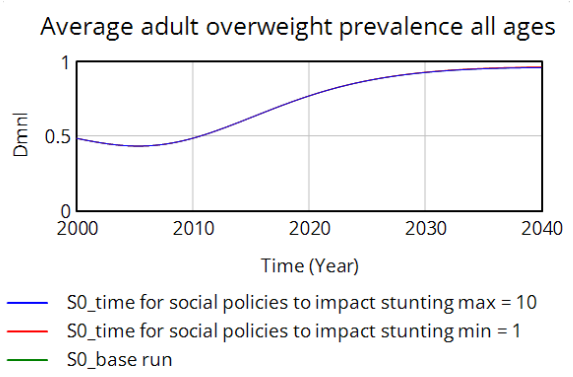

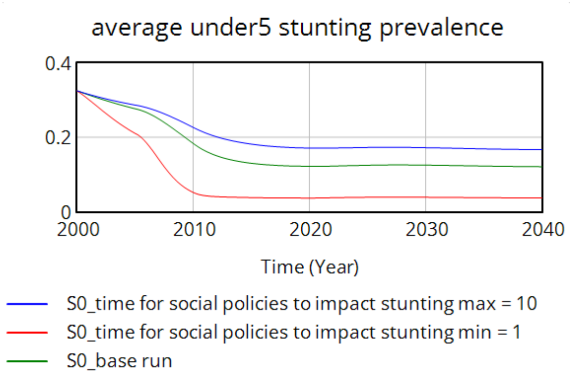
</td><td>Yes</td></tr>
</table>

| Variable | Description | Simulation results | Simulations as expected |
|---|---|---|---|
| Political will per policy ( = 10 1/policy) | "Political will per policy" variable represents the amount of policy efforts that can be achieved against overweight and stunting. It is interpreted relatively to the variable "Political will for nutrition policy", which takes a value of 100, so it can be interpreted as 10% of total political will is required to achieve one policy.<br><br>We varied "Political will per policy" to take values min = 1, i.e. it takes 1% of total political will to achieve one policy, so 10 times more policies can be achieved compared to the base run, and max = 100, i.e. it takes 100% of total political will to achieve one policy, so only 1 policy can be achieved, which is 10 times less that in the base run. Under these extreme scenarios, we expect that overweight will only very slightly change, as under the Baseline scenario policy only slightly impacts overweight. We expect under-5 stunting to decrease faster (for min) and slower (for max) compared to the base run, as the efforts for its reduction through policy will be much higher and lower respectively. | 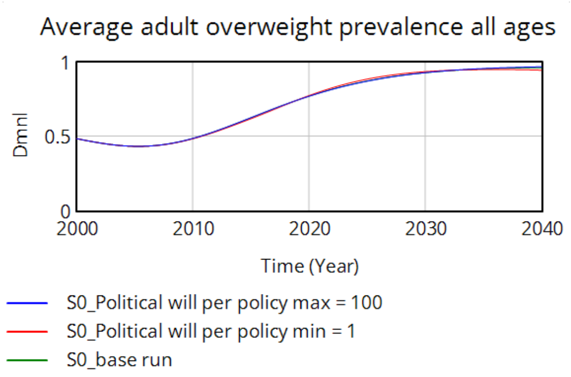<br>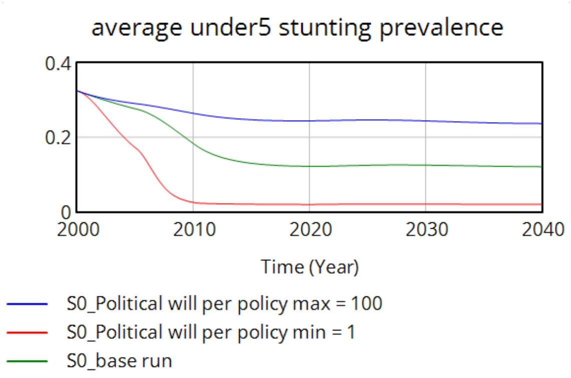 | Yes |

| Variable | Description | Simulation results | Simulations as expected |
|---|---|---|---|
| effect of social policies on stunting (= -0.2 1/policy) | "effect of social policies on stunting" is based on an assumption, calibrated against observed stunting prevalence. SA showed that under-5 stunting prevalence is numerically sensitive to changes in this variable. We varied "effect of social policies on stunting" to take values min = 0, i.e. social policies have no effect on stunting, and max = 1, i.e. social policies reduce stunting by 100% per policy. Under these extreme scenarios, we expect that overweight will not change, as social policies do not impact overweight in this model. We expect under-5 stunting to not decrease significantly (for min), as social policies is the main driver of stunting reduction in the Baseline scenario, and faster (for max) compared to the base run, as social policies will be more effective. | 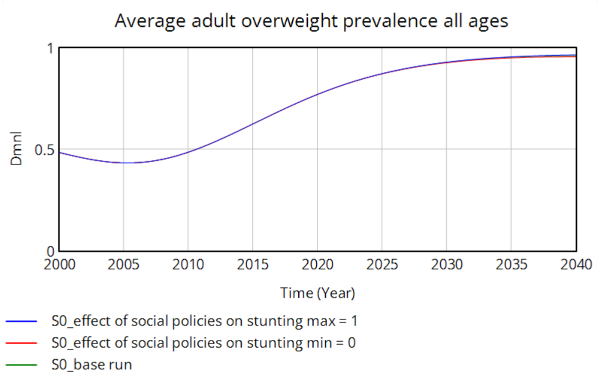<br>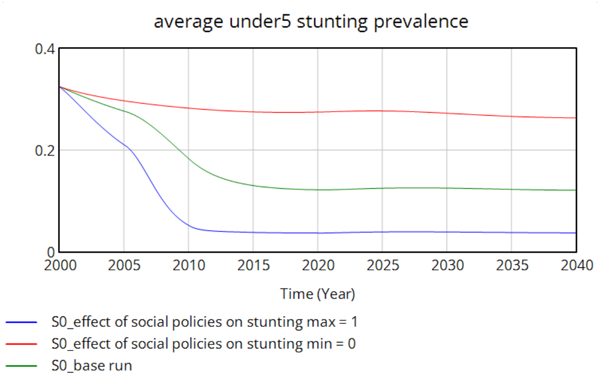 | Yes |
| Mortality rates | | | Yes |

| Variable | Description | Simulation results | Simulations as expected |
| --- | --- | --- | --- |
| | Mortality rates at baseline and its change over time for all age groups was obtained from data from the Instituto Nacional de Estadística e Informática (INEI) of Peru. We varied for variables for this extreme scenario: Baseline child mortality rate, child mortality rate annual change, Baseline adult mortality rate and adult mortality rate annual change. For the minimum extreme scenario, we assumed that all these variables were zero, i.e. no population died. For the maximum extreme scenario, we assumed that baseline mortality variables are 1, with no further change in mortality during the simulation. Under these scenarios, we assume that population will not notably reduce (min mortality) or it will quickly reach zero (max mortality). This will result in similar effects in overall overweight and stunted population, although overweight and stunting prevalence will not change, as they are the ratio of the two populations. | 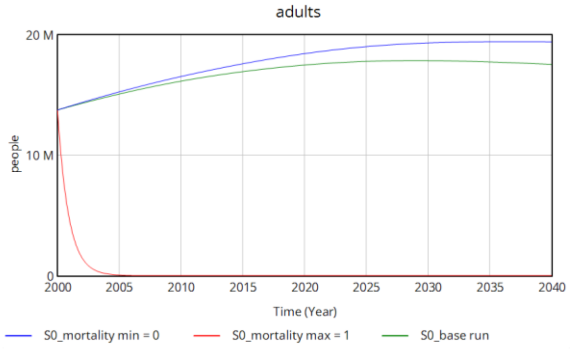<br>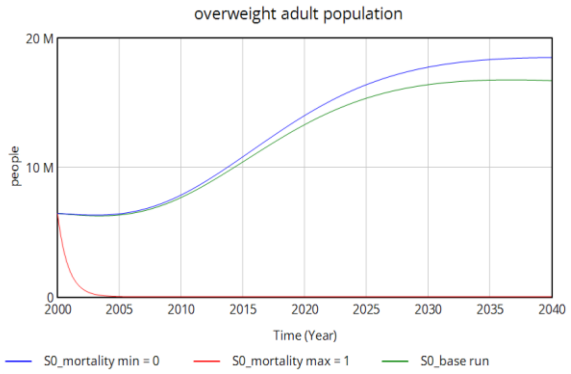 | |

<table>
<tr><th>Variable</th><th>Description</th><th>Simulation results</th><th>Simulations as expected</th></tr>
<tr><td></td><td></td><td>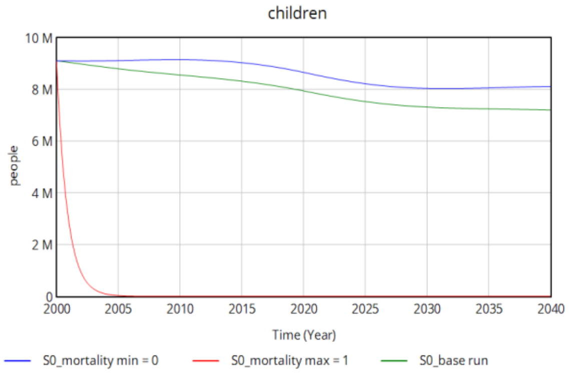

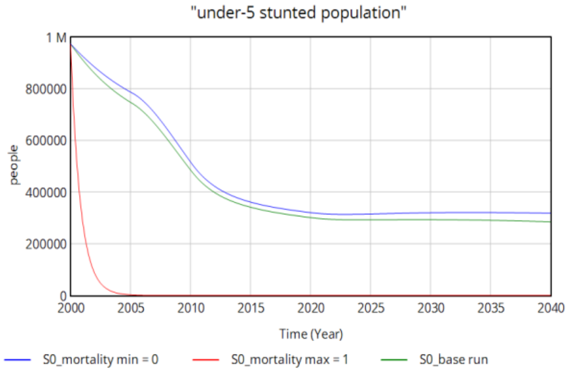
</td><td></td></tr>
</table>

Conclusion

- For extreme variations of different variables that are important to the behaviour of the model, namely “Industry goal for UPEP consumption”, “Effect of UPEP on overnutrition”, “time for diet to impact nutrition outcomes”, “Time for social policies to impact stunting”, “Political will per policy”, and “effect of social policies on stunting”, Average adult overweight and Average under-5 stunting variables behave as expected.
- For extreme variations of variables that impact the aging chains, such as child mortality rate and adult mortality rate, overall child and adult population stocks as well as overweight and stunted populations behave as expected. To facilitate this, we added death rate outflows to overweight and stunting stocks for both children and adults.

**Supplementary Table S1. Table with variables used in the model. The variable names, units of measure, value (for constants) and equations (for all other variables), brief description, and source, where relevant, are presented. The variables are presented by type of variable which include: Constants, Auxiliaries, Stocks, Flow, and Smooth variables.**

| Variable name | Units | Value or Equation | Description | Source (if any) |
|---|---|---|---|---|
| *Constants (N=68)* | | | | |
| Added delay to achieve desired consumption | Year/servings | 1 | Assumption: For each serving increase wanted to achieve, the industry will double their initial delay due to lobbying | |
| adult cohort duration [adult age] | Year | 10,10,15 | Duration of each age group: 15-24, 25-34, 35-49 | |
| adult mortality rate annual change [adult age] | 1/Year/Year | -2.13333e-05,-3.16667e-05,-6e-05 | Estimated as annual mortality rate change between 2000 and 2020 (mean of 5-year age groups) | INEI[6] |
| Baseline adult mortality rate [adult age] | 1/Year | 0.00137,0.002157,0.00454 | Mean estimated mortality rates of 5-year age groups in 2000 | INEI[6] |
| Baseline adult overweight prevalence [adult age] | Dmnl | 0.28143,0.505714,0.663629 | Prevalence of overweight (BMI>=25) in women 20-49 in Peru, 2000. Note: No data on men available. For women 15-19, BMI is not appropriate to estimate overweight and no data on z-score are available | ENDES, 2000[8] |
| Baseline child mortality rate | 1/Year | Baseline child mortality rate[child age] = 0.0159133,0.000855,0.0008 | Mean estimated mortality rates of 5-year age groups in 2000 | INEI[6] |
| baseline child ow prevalence [child age] | Dmnl | 0.118613,0.211436,0.215506 | Age 0 to 4: Prevalence of overweight (weight for height z-score>2SD) in children under-5 in Peru, 2000. Estimated from ENDES, 2000<br>Ages 5 to 9 and 10 to 14: Prevalence of overweight (BMI-age z-score>1SD) in children and adolescents in Peru in 2000 (average of prevalence by 1 year of age) - modelled estimates. | Ages 0 to 4: ENDES, 2000[8]<br>Ages 5 to 9 and 10 to 14: NCD Risk Factor Collaboration[9]. |
| baseline child stunting prevalence [child age] | Dmnl | 0.325,0.2761,0.2761 | 0to4: Stunting prevalence (height for age z-score<-2SD) for children under-1 in Peru in 2000, Estimated from ENDES, 2000<br>5to9 and 10to14: Stunting prevalence among children aged 8 in 2001. Estimated from Young Lives cohort (Round 1, old cohort) NOTE: This study is not nationally representative and it over-represents deprived households. | Ages 0 to 4: ENDES, 2000[8]<br>Ages 5to9 and 10to14: Young Lives[10] |
| Baseline fertility rate [adult age] | 1/Year | 0.103,0.123,0.039 | Fertility rate (births per woman) by age group (mean of 5-year age groups) | ENDES, 2000[8] |

| | | | | |
|---|---|---|---|---|
| Baseline infant overweight prevalence | Dmnl | 0.1333 | Overweight prevalence (weight for height z-score>2SD) for children under-1 in Peru in 2000 | ENDES, 2000[8] |
| Baseline low birth weight | Dmnl | 0.0688 | Estimated from 2015 low birth weight national birth registry data (prevalence = 6.4%), adjusted for 2000 using modelled trends of low birth weight in Peru between 2000 and 2015 (prevalence change = -7.45%) | 2015 data: Carrillo-Larco et al, 2021[11]. Trend data: Blencowe et al, 2019[12] |
| baseline population adults [adult age] | people | 5.26445e+06,4.20224e+06,4.27832e+06 | Total population by age group in 2000 | INEI[13] |
| baseline population children [child age] | people | 2.99496e+06,3.0832e+06,3.02156e+06 | Total population by age group in 2000 | INEI[13] |
| baseline short stature prevalence [adult age] | Dmnl | 0.106488, 0.121711, 0.170064 | Prevalence of short stature (height<145cm) in women in Peru, 2000. Note: no data on men | ENDES, 2000[8] |
| change in price due to inflation | Dmnl | 0.03 | Inflation, consumer prices (annual %) in Peru, average between 2000-2022. | World Bank |
| child cohort duration | Year | 5 | duration of each age group: 0-4, 5-9, 10-14 | |
| child mortality rate annual change [child age] | 1/Year/Year | -0.000466833,-1.625e-05,-1.325e-05 | Estimated as annual mortality rate change between 2000 and 2020 (mean of 5-year age groups) | INEI[6] |
| distribution of resources for production vs marketing | Dmnl | 0.5 | Assumption: We assume that the industry splits its efforts to increase profits equally between increasing production and marketing | |
| Double-duty policy nutrition education prc | Dmnl | 0.1 | Assumption: 10% of double-duty policies focus on nutrition education – used only under Scenario 6 | |
| effect of marketing on acceptability | Dmnl | 0.1 | Children exposed to unhealthy foods/beverages marketing had a higher risk of selecting the advertised products that were associated with a familiar licenced-character/logo (RR = 1.1, 95%CI 1.0 to 1.2; P = 0.052) | Sadeghirad, et al, 2016[14] |
| effect of MPF on stunting | 1/servings | -0.001 | Assumption: It is assumed that there is a very small (0.1%) reduction in stunting for each serving of healthy food consumed. This is consistent with evidence that shows that child nutrition did not play a significant role in reducing stunting in Peru (Huicho et al, 2020) | Huicho et al, 2020[15] |

| | | | | |
|---|---|---|---|---|
| Effect of nutrition education on acceptability | 1/policy | 0.0001 | Assumption: It is assumed that nutrition education programmes have a very small effect (0.01%) on MPF acceptability. This is based on evidence on the pathways to improve nutrition through social protection programmes in the LAC region, which showed that the education component of such programmes did not have an effect on nutrition knowledge. | Nisbett et al, 2022[16]. |
| Effect of policy on UPEP availability | servings/policy | -0.1 | Assumption: It is assumed that for each overnutrition policy, there is a 10% reduction in availability of UPEP | |
| effect of social policies on stunting | 1/policy | -0.2 | Assumption: It is assumed that there is a 20% reduction in stunting due to social policies. This corresponds to observed reduction in stunting between 2000 and 2015, which was mainly due to social policies, according to Huicho et al. | Huicho et al, 2020[15] |
| effect of UPEP on overnutrition | 1/servings | 0.06 | A meta-analysis of observational studies has shown a RR=1.18 (95% CI: 1.09, 1.27, $p < 0.001$) for highest vs lowest consumers of UPF. If we assume that consumption of UPF ranges between 0 and 3 servings (with initial=1), then effect per serving is (1.18 - 1)/3 = 0.06 | Askari et al, 2020[17] |
| effect of warning labels on acceptability | Dmnl | -0.24 | The likelihood of purchasing a product without a warning label was 2.59 compared to the likelihood of purchasing a product with an octagon, which was 1.97, a 24% reduction | Taillie et al, 2020 [18] |
| elasticity of demand MPF | Dmnl | -0.7475 | Average own-price elasticity for 4 food groups (fruit whole grains, milk, fish) in Latin America and the Caribbean | Muhammad et al, 2017[19] |
| elasticity of demand UPEP | Dmnl | -0.24 | Average own-price elasticity for 3 food groups (Processed red meat, SSB, Fruit juice) in Latin America and the Caribbean | Muhammad et al, 2017[19] |
| elasticity of supply | Dmnl | -0.1 | Assumptions: Food supply is inelastic. Elasticity of supply is the same for UPEP and MPF | |
| female percentage | Dmnl | 0.501 | Population, female (% of total population), Peru, 2000 | World Bank |
| fertility rate annual change [adult age] | 1/Year/Year | -0.002175,-0.0016,-0.00055 | Estimated as annual fertility rate change between 2000 and 2020 (mean of 5-year age groups) | ENDES, 2000[8] ENDES, 2020[20] |

| | | | | |
|---|---|---|---|---|
| Habit replacement time | Year | 5 | Assumption: It takes 5 years for people to change the habit of consuming UPEP and MPF. This assumption is based on inputs from stakeholders who argued that policies often fail because food habits/culture are built over time and they are not easy to change. We performed sensitivity analyses to further explore the validity of this variable | |
| Industry goal for UPEP consumption | servings | 3 | Latest data from 2019 show that UPF consumption in Chile, which is the country with highest UPF consumption, is three times higher than this in Peru. So we assume that the industry's goal is for Peru to reach the max consumption in the region, i.e. 3 times more than the initial consumption (1 serving) | PAHO, 2019[21] |
| Initial food from food assistance | servings | 0.14 | Average number of servings received every time a household received food assistance in 2000 | ENAHO, 2000[22] |
| Initial MPF consumption | servings | 9 | Estimated as total consumption of healthy (minimally-processed) food (MPF) excluding consumption of unhealthy (ultra-processed) food in Peru in 2000 | Global Dietary Database, Peru, 2000[23] |
| Initial percentage of UPEP targeted by labelling | 1/policy | 0.842 | 15.8% of UPEP examined had no octagons, 6 months after the implementation of the law. | Meza-Hernández et al, 2020[24] |
| Initial undernutrition policies | policy | 2 | Assumption: There are 2 undernutrition policies at baseline. This is based on Peru's policy efforts during the 90s and early 00s that mainly focused on food assistance initiatives under the National Program for Food Assistance (PRONAA) and the Glass of Milk program. | Mejia Acosta et al, 2014[25] |
| Initial UPEP consumption | servings | 1 | Estimated as consumption of three food groups (Total processed meats, Sugar-sweetened beverages, and Fruit juices) in Peru in 2000. This estimate is consistent with reports that want UPF to constitute approx. 20% of food sales in Peru, given that total food intake assumed here is 10 servings (MPF+UPEP). | Global Dietary Database, Peru, 2000[23] |
| Lifespan of nutrition policies | Year | 5 | Assumption: The lifespan of an active nutrition policy is 5 years and equals to the amount of time needed for policy implementation | |
| low birth weight and stunting RR | Dmnl | 3.67 | Regional odds ratio for stunting at 12 to 60 months among babies born low birthweight in Latin America from a meta-analysis of birth cohorts. | Christian et al, 2013[26] |

| | | | | |
|---|---|---|---|---|
| maternal height and low birth weight RR | Dmnl | 1.98 | Pooled adjusted RRs between short maternal stature (<145 cm, reference: >=155 cm) and short for gestational age (<10% compared to >=10%) | Kozuki et al, 2015 [27] |
| overnutrition goal | Dmnl | 0.6 | Informed by the 2025 Global nutrition target for overweight and obesity: No increase in overweight and obesity. We assume that the target is interpreted as a goal of 60% overweight, which is approximately the average prevalence of overweight in women in 2015, approximately when the global nutrition targets were implemented | WHO[28] |
| Overnutrition policy availability prc | Dmnl | 0.25 | Percentage of overnutrition policies that target UPEP availability (e.g. school kiosk programmes). We assume that at baseline these are 25% of total overnutrition policies. | |
| Overnutrition policy marketing prc | Dmnl | 0.25 | Percentage of overnutrition policies that target UPEP marketing (e.g. ban on advertising). We assume that at baseline these are 25% of total overnutrition policies. | |
| Overnutrition policy nutrition education prc | Dmnl | 0.25 | Percentage of overnutrition policies that are nutrition education. We assume that at baseline these are 25% of total overnutrition policies. | |
| percentage of UPEP targeted by marketing | Dmnl | 0.7 | According to an analysis of marketing techniques on processed foods and beverages in Lima, Peru, almost seven out of ten products (70%) displayed at least one of the ten marketing techniques investigated. | Saavedra-Garcia et al, 2022[29] |
| Political will for nutrition policy | Dmnl | 100 | The total (100%) political will that exists for nutrition policy | |
| Political will per policy | 1/policy | 10 | Assumption: 10% of total political will is needed to implement one policy | |
| time for diet to impact nutrition outcomes | Year | 1 | Assumption: it takes 1 year for a change in diet to reflect a change in nutrition outcomes | |
| time for industry to achieve goal | Year | 5 | Assumption: The industry adjusts its sales goal gradually over a 5-year period | |
| Time for policy implementation | Year | 5 | Assumption: It takes 5 years for a policy to be implemented and it equals to the lifespan of nutrition policies | |
| time for social policies to impact stunting | Year | 5 | Assumption: It takes 5 years for social policies to have an effect on stunting | |
| Time to change acceptability | Year | 1 | Assumption: It takes 1 year to change UPEP preference of a population | |

| | | | | |
|---|---|---|---|---|
| undernutrition goal | Dmnl | 0.09 | 2025 Global nutrition targets: 40% reduction in childhood stunting<br>Assumption: We assume that the target is interpreted as a goal of 9% stunting, which is approximately 40% lower than the achieved 14.7% in 2015 | WHO[28] |
| undernutrition policy monitoring | Dmnl | 1 | Assumption: Undernutrition policy monitoring is present in full capacity since 2000 | |
| Undernutrition policy nutrition education prc | Dmnl | 0.1 | Percentage of undernutrition policies that are nutrition education<br>Assumption: 10% of undernutrition policies are nutrition education | |
| UPEP price change per policy | 1/policy | 0.15 | We assume that a price related policy will increase prices of UPEP by 15% | |
| S1 | Dmnl | 0 | Scenario variable<br>Scenario 1: Introduction of price policies: In 2015, 25% of overnutrition policies will be price policies, e.g. taxes, that aim to increase price of UPEP - For Scenario 1, change from 0 to 0.25 | |
| S2a | Year | 7 | Scenario variable<br>S2: S1 + Reduce policy delay, i.e. the time between political will and policy action for both overnutrition and undernutrition: In 2015, time between political will and action for overweight policy drops by 7 years, i.e. from 8 years to 1 year - For Scenario 2, change from 0 to 7 | |
| S2b | Year | 7 | Scenario variable<br>S2: S1 + Reduce policy delay, i.e. the time between political will and policy action for both overnutrition and undernutrition: In 2015, time between political will and action for undernutrition policy drops by 7 years, i.e. from 8 years to 1 year. - For Scenario 2, change from 0 to 7 | |
| S3 | Dmnl | 0 | Scenario variable<br>S3: S2 + Increase overweight monitoring: Overnutrition policy success is as well monitored as undernutrition policy success - For Scenario 3, change from 0 to 0.5 | |

| | | | | |
|---|---|---|---|---|
| S4 | Dmnl | 1 | Scenario variable<br>S4: S3 + Regulate quality of foods available through undernutrition policies: No UPF can be provided through undernutrition policies, such as food assistance programmes. When this variable is zero, there is no UPEP from food assistance policies - For Scenario 4, change from 1 to 0 | |
| S5 | Year | 0 | Scenario variable<br>S5: S4 + Regulate industry lobbying: Industry plays no role in the overnutrition policy making process - For scenario 5, change from 0 to 6 | |
| S6 | Dmnl | 0 | Scenario variable<br>S6: S5 + Double-duty policies informed by DBM progress When S6 is 1, it means that nutrition policy does not rely on prioritisation of undernutrition over overnutrition and that double-duty policies are implemented instead - For Scenario 6, change from 0 to 1 | |
| *Auxiliary (Endogenous) variables (N=52)* | | | | |
| adult mortality rate [adult age] | 1/Year | Baseline adult mortality rate[ adult age]+RAMP( adult mortality rate annual change[ adult age], INITIAL TIME, INITIAL TIME+20) | Assumption: Mortality rate changes between 2000 and 2020, according to observed data. After 2020, it does not change. | |
| adults | people | SUM( adults sb[ adult age!]) | Total number of adults - for validation purposes | |
| Average adult overweight prevalence [adult age] | Dmnl | overweight adults[ adult age]/ adults sb[ adult age] | Prevalence is estimated as the number of people with the characteristic of interest divided by the total amount of people | |
| Average adult overweight prevalence all ages | Dmnl | SUM( Average adult overweight prevalence[ adult age!])/3 | | |
| Average child overweight prevalence [child age] | Dmnl | overweight children[ child age]/ children sb[ child age] | Prevalence is estimated as the number of people with the characteristic of interest divided by the total amount of people | |
| average short stature prevalence [adult age] | Dmnl | short stature adults[ adult age]/ adults sb[ adult age] | Prevalence is estimated as the number of people with the characteristic of interest divided by the total amount of people | |

| | | | | |
|---|---|---|---|---|
| average stunting prevalence [child age] | Dmnl | stunted children[ child age]/ children sb[ child age] | Prevalence is estimated as the number of people with the characteristic of interest divided by the total amount of people | |
| Change in acceptability because of nutrition education | servings | MPF acceptability* Effect of nutrition education on acceptability* Nutrition Education | | |
| change in acceptability due to marketing | servings | change in products targeted by marketing* effect of marketing on acceptability | | |
| change in acceptability due to labelling | servings | products targeted by labelling *effect of warning labels on acceptability | | |
| Change in MPF availability | servings | (( Active undernutrition policies- Initial undernutrition policies)* Undernutrition policy food assistance prc* MPF from food assistance per policy)+( Active Double-duty policies* Double-duty policy availability prc* MPF from food assistance per policy) | Change in MPF availability depends on food provision policies (e.g. food assistance programmes) | |
| change in MPF price | Dmnl | (Change in MPF availability/ Initial MPF consumption)* elasticity of supply | Price changes as availability (i.e. supply) changes, according to the definition of price elasticity, % change in quantity supplied = % change in price * price elasticity of supply | |
| change in products targeted by marketing | servings | Gap between actual and industry desired UPEP consumption* percentage of UPEP targeted by marketing* distribution of resources for production vs marketing | | |

| | | | | |
|---|---|---|---|---|
| Change in UPEP availability | servings | change in UPEP production+( Active overnutrition policies* Overnutrition policy availability prc* Effect of policy on UPEP availability)+(( Active undernutrition policies- Initial undernutrition policies)* Undernutrition policy food assistance prc* UPEP per food assistance policy)+( Active Double-duty policies* Undernutrition policy food assistance prc* UPEP per food assistance policy)+( Active Double-duty policies* Double-duty policy availability prc* Effect of policy on UPEP availability) | Change in UPEP availability depends on: 1. Changes in UPEP production due to industry activity, 2. Policies that reduce UPEP availability (e.g. school kiosk programmes), 3. Food provision policies (e.g. food assistance programmes) | |
| Change in UPEP consumption through acceptability | servings | UPEP acceptability- Initial UPEP consumption | | |
| Change in UPEP consumption through price | servings | change in UPEP price* elasticity of demand UPEP* UPEP consumption habit | Change in price according to the definition of price elasticity, % change in quantity demanded = % change in price * price elasticity of demand | |
| change in UPEP price | Dmnl | (( Change in UPEP availability/ Initial UPEP consumption)* elasticity of supply)+ change in price due to inflation+( Active overnutrition policies* Overnutrition policy price prc* UPEP price change per policy)+( Active Double-duty policies* Double-duty policy price prc* UPEP price change per policy) | Based on the definition of price elasticity, % change in quantity supplied = % change in price * price elasticity of supply - We divide change in availability with initial to estimate percentage change | |
| change in UPEP production | servings | Gap between actual and industry desired UPEP consumption* distribution of resources for production vs marketing | UPEP production changes to reduce the gap between their industry desired consumption and actual UPEP consumption | |
| child mortality rate [child age] | 1/Year | Baseline child mortality rate[ child age]+RAMP( child mortality rate annual change[ child age], INITIAL TIME, INITIAL TIME+20) | Assumption: Mortality rate changes between 2000 and 2020, according to observed data. After 2020, it does not change. | |
| children | people | SUM( children sb[ child age!]) | Total number of children - for validation purposes | |

| | | | | |
|---|---|---|---|---|
| DBM gap | Dmnl | (Average adult overweight prevalence all ages- overnutrition goal) + (average stunting prevalence[A0to4]- undernutrition goal) | The distance of overweight and stutning prevalence from their respective goals | |
| Delay due to lobbying | Year | Normal delay due to lobbying+( Gap between actual and industry desired UPEP consumption* Added delay to achieve desired consumption) | Delay between political will and overnutrition policy caused due to industry lobbying. The larger the gap between industry desired consumption and actual consumption of UPEP, the larger the delay due to more intense industry efforts | |
| Double-duty policy availability prc | Dmnl | (1- Double-duty policy nutrition education prc- Undernutrition policy - social policy prc)/3 | Assumption: A third of remaining double-duty policies (excluding social policies and nutrition education) are availability policies. The other are price policies and marketing policies | |
| Double-duty policy marketing prc | Dmnl | (1- Double-duty policy nutrition education prc- Undernutrition policy - social policy prc)/3 | Assumption: A third of remaining double-duty policies (excluding social policies and nutrition education) are marketing policies. The other are price policies and availability policies | |
| Double-duty policy price prc | Dmnl | (1- Double-duty policy nutrition education prc- Undernutrition policy - social policy prc)/3 | Assumption: A third of remaining double-duty policies (excluding social policies and nutrition education) are price policies. The other are availability policies and marketing policies | |
| fertility rate [adult age] | 1/Year | Baseline fertility rate[ adult age]+RAMP( fertility rate annual change[ adult age], INITIAL TIME, INITIAL TIME+20) | Assumption: Fertility rate changes between 2000 and 2020, according to observed data. After 2020, it does not change. | |
| Food from food assistance per policy | servings/policy | Initial food from food assistance/( Initial undernutrition policies* Initial undernutrition policy food assistance prc) | Food provided from food assistance policies per policy is estimated from total food from food assistance in 2000, divided by the total number of food assistance policies in 2000 Assumption: Food per food assistance policy does not change over time | |
| Gap between actual and industry desired UPEP consumption | servings | Industry desired UPEP consumption- UPEP consumption habit | The difference between industry desired and actual UPEP consumption | |
| Initial undernutrition policy food assistance prc | Dmnl | INITIAL( Undernutrition policy food assistance prc) | Percentage of undernutrition policy that is food provision policy, e.g. food assistance programmes at the start of the simulation period | |
| Initial products targeted by labelling per policy | servings/policy | Initial percentage of UPEP targeted by labelling * Initial UPEP consumption | | |
| low birth weight rate [adult age] | Dmnl | (( Baseline low birth weight*(1- average short stature prevalence[ adult age]))+( Baseline low birth weight* average short stature prevalence[ adult age]* maternal height and low birth weight RR)) | Low birth weight rate (for each age group) is estimated as the birth weight at baseline from adults with no short stature plus birth weight at baseline from adults with short stature. The latter will change as adults with short stature change, with effect estimate informed by relevant relative risk (RR) | |

| | | | | |
|---|---|---|---|---|
| MPF from food assistance per policy | servings/policy | Food from food assistance per policy*(1- UPEP consumption percent) | MPF from food assistance equals to total food from food assistance after excluding UPEP | |
| Normal delay due to lobbying | Year | 6 - STEP(S5, 2015) | Assumption: At baseline, the normal delay to policy action due to lobbying is 6 years and is equal to the amount of time between initial discussions for the food advertising law in 2013 until its initial implementation in 2019. Under Scenario 5, this drops by S5 in 2015. | |
| Normal time between political will and action for overnutrition policy | Year | 8 - STEP( S2a,2015) | Assumption: At baseline, the normal time between political will and action for overnutrition policy if there was no industry involvement equals to the time between political will for action and undernutrition policy. Under Scenario 2, this drops by S2a in 2015. | |
| Nutrition Education | policy | ( Active overnutrition policies* Overnutrition policy nutrition education prc)+( Active undernutrition policies* Undernutrition policy nutrition education prc)+( Active Double-duty policies* Double-duty policy nutrition education prc) | | |
| overnutrition policy monitoring | Dmnl | IF THEN ELSE(Average adult overweight prevalence all ages>0.6, 0.5 + S3 , 0) | Assumption: Until adult overweight prevalence reaches 60%, there is no monitoring of overnutrition policy. When it reaches 60%, monitoring starts at half capacity of the undernutrition policy monitoring (at Baseline Scenario - S0) or full monitoring policy under Scenario 3 | |
| Overnutrition policy price prc | Dmnl | 0 + STEP( S1,2015) | Percentage of overnutrition policies that target UPEP price (e.g. sugar taxes). Assumption: There are no price policies at baseline. Under Scenario 1, there are S1 policies implemented in 2015 | |
| overnutrition policy success | Dmnl | (1- Average adult overweight prevalence all ages)* overnutrition policy monitoring | The more overweight prevalence reduces, the larger the overnutrition policy success | |
| overweight change rate | 1/Year | (( UPEP consumption habit- Initial UPEP consumption)* effect of UPEP on overnutrition)/ time for diet to impact nutrition outcomes | Overweight changes due to changes in UPEP consumption | |
| Political will for overnutrition policy | Dmnl | ( Political will for nutrition policy*MAX((1- Prioritisation of undernutrition over overnutrition)*0.5,0))*(1-STEP( S6,2015)) | At baseline, political will for overnutrition policy depends on will for nutrition policy and prioritisation of undernutrition over overnutrition. The highest the prioritisation of undernutrition, the lower the percentage of overall will that is dedicated to overnutrition. We multiply by 0.5, so that will for overnutrition+will for undernutrition is never more than 1. We use the MAX function so that percentage is never lower than 0. | |
| Political will for undernutrition policy | Dmnl | ( Political will for nutrition policy*MIN((1+ Prioritisation of undernutrition over overnutrition)*0.5,1))*(1-STEP( S6,2015)) | At baseline, political will for undernutrition policy depends on will for nutrition policy and prioritisation of undernutrition over overnutrition. The highest the prioritisation of undernutrition, the higher the percentage of overall will that is dedicated to undernutrition. We multiply by 0.5, so that will for overnutrition+will for undernutrition is | |

| | | | | |
|---|---|---|---|---|
| | | | never more than 1. We use the MIN function so that percentage is never higher than 1. | |
| Prioritisation of undernutrition over overnutrition | Dmnl | IF THEN ELSE( overnutrition policy success=0,1, undernutrition policy success- overnutrition policy success) | The higher the undernutrition policy success and/or the lower the overnutrition policy success, the higher the prioritisation of undernutrition over overnutrition | |
| products targeted by labelling | servings | (Initial products targeted by labelling per policy * Active overnutrition policies * Overnutrition policy marketing prc) + (Initial products targeted by labelling per policy * "Active Double-duty policies" * "Double-duty policy marketing prc") | Products targeted by labelling change as policies change | |
| stunting change rate | 1/Year | ((( MPF consumption habit- Initial MPF consumption)* effect of MPF on stunting)/ time for diet to impact nutrition outcomes)+ stunting change rate social policies | Stunting changes due to changes in MPF consumption and impacts of social policies | |
| stunting change rate social policies | 1/Year | (( Active undernutrition policies* Undernutrition policy - social policy prc* effect of social policies on stunting)+( Active Double-duty policies* Undernutrition policy - social policy prc* effect of social policies on stunting))/ time for social policies to impact stunting | Change in stunting due to social policies | |
| Time between political will and action for overnutrition policy | Year | Normal time between political will and action for overnutrition policy+ Delay due to lobbying | The actual time it takes for political will to become action is the normal time it takes for political will to become action plus the delay caused by industry lobbying | |
| Time between political will and action for undernutrition policy | Year | 8 - STEP( S2b,2015) | Assumption: At baseline, we assume that it takes 8 year for political will for undernutrition policy to become action.Based on the time between the pilot phase of JUNTOS in 2005 (coverage of 1.4% of rural families) to its scale-up in 2013 (coverage 10.1% of rural families). Under Scenario 2, this drops by S2b in 2015. | Huicho et al, 2016[30] |
| Undernutrition policy - social policy prc | Dmnl | 0.1 + RAMP(0.1, 2005, 2010) | Percentage of undernutrition policy that is social policy. Baseline scenario: In 2000 there were almost no social policies affecting undernutrition. In 2005 Juntos was gradually introduced. Social | Huicho et al, 2016[30] |

| | | | | |
|---|---|---|---|---|
| | | | policies were increasing until 2010, when there was the introduction of cross-cutting antipoverty and social inclusion policies | |
| Undernutrition policy food assistance prc | Dmnl | 1- Undernutrition policy - social policy prc- Undernutrition policy nutrition education prc | Percentage of undernutrition policy that is food provision policy, e.g. food assistance programmes | |
| undernutrition policy success | Dmnl | (1- average stunting prevalence[A0to4])* undernutrition policy monitoring | The more stunting prevalence reduces, the larger the overnutrition policy success | |
| UPEP consumption percent | Dmnl | UPEP consumption habit/( MPF consumption habit+ UPEP consumption habit) | | |
| UPEP per food assistance policy | servings/policy | Food from food assistance per policy* UPEP consumption percent*STEP( S4,2015) | | |
| *Stocks (N=14)* | | | | |
| Active Double-duty policies | policy | ∫New double-duty policies- End of double-duty policies dt + 0.0 | | |
| Active overnutrition policies | policy | ∫New overnutrition policies- End of overnutrition policies dt + 0.0 | | |
| Active undernutrition policies | policy | ∫ New undernutrition policies- End of undernutrition policies dt + Initial undernutrition policies | | |
| Industry desired UPEP consumption | servings | ∫ change in industry desired consumption dt + Initial UPEP consumption | Assuming that initially industry desired UPEP consumption equals to actual UPEP consumption | |
| MPF acceptability | servings | ∫- change in acceptability dt + Initial MPF consumption | | |
| MPF consumption habit | servings | ∫change in MPF consumption dt + Initial MPF consumption | | |
| UPEP acceptability | servings | ∫change in acceptability dt + Initial UPEP consumption | | |
| UPEP consumption habit | servings | ∫ change in UPEP consumption dt + Initial UPEP consumption | | |

| | | | | |
|---|---|---|---|---|
| adults sb [adult age] | people | ∫( child aging[A10to14]- adult aging[A15to24])- adult death rate[A15to24] dt + baseline population adults[A15to24]<br>∫ ( adult aging[A15to24]- adult aging[A25to34])- adult death rate[A25to34] dt + baseline population adults[A25to34]<br>∫(adult aging[A25to34]- adult death rate[A35to49])- adult aging[A35to49] *dt* + baseline population adults[A35to49] | adult population stock with subscripts (i.e. by age group) in increasing order of age | |
| children sb [child age] | people | ∫ ( births- child aging[A5to9])- child death rate[A0to4] *dt* + baseline population children[A0to4]<br>∫ ( child aging[A0to4]- child death rate[A5to9])- child aging[A5to9] *dt* + baseline population children[A5to9]<br>∫ ( child aging[A5to9]- child death rate[A10to14])- child aging[A10to14] *dt* + baseline population children[A10to14] | children population stock with subscripts (i.e. by age group) in increasing order of age | |
| overweight adults [adult age] | people | ∫ (( overweight aging[A10to14]- adult overweight aging[A15to24])+ adult overweight change[A15to24])- ow adult death rate[A15to24] dt + Baseline adult overweight prevalence[A15to24]* baseline population adults[A15to24]<br>∫ (( adult overweight aging[A15to24]- adult overweight aging[A25to34])+ adult overweight change[A25to34])- ow adult death rate[A25to34] dt + Baseline adult overweight prevalence[A25to34]* baseline population adults[A25to34] | Overweight adult population stock with subscripts (i.e. by age group) in increasing order of age | |

| | | | | |
|---|---|---|---|---|
| | | ∫ (( adult overweight aging[A25to34]- adult overweight aging[A35to49])+ adult overweight change[A35to49])- ow adult death rate[A35to49] dt + Baseline adult overweight prevalence[A35to49]* baseline population adults[A35to49] | | |
| overweight children [child age] | people | ∫ (( overweight births- overweight aging[A0to4])+ child overweight change[A0to4])- ow children death rate[A0to4] dt + baseline child ow prevalence[A0to4]* baseline population children[A0to4] | Overweight child population stock with subscripts (i.e. by age group) in increasing order of age | |
| | | ∫ (( overweight aging[A0to4]- overweight aging[A5to9])+ child overweight change[A5to9])- ow children death rate[A5to9] dt + baseline child ow prevalence[A5to9]* baseline population children[A5to9] | | |
| | | ∫ (( overweight aging[A5to9]- overweight aging[A10to14])+ child overweight change[A10to14])- ow children death rate[A10to14] dt + baseline child ow prevalence[A10to14]* baseline population children[A10to14] | | |
| short stature adults | people | ∫ ( stunted aging[A10to14]- short stature aging[A15to24])- short stature adult death rate[A15to24] dt + baseline short stature prevalence[A15to24]* baseline population adults[A15to24] | Adult short stature population stock with subscripts (i.e. by age group) in increasing order of age | |
| | | ∫ ( short stature aging[A15to24]- short stature aging[A25to34])- short stature adult death rate[A25to34] dt + baseline short stature prevalence[A25to34]* baseline population adults[A25to34] | | |
| | | ∫ ( short stature aging[A25to34]- short stature aging[A35to49])- short stature adult death rate[A35to49] dt + baseline short stature prevalence[A35to49]* baseline population adults[A35to49] | | |
| stunted children [child age] | people | ∫ (((SUM( stunted births[ adult age!]))- stunted aging[A0to4])+ stunting change[A0to4])- stunted children death rate[A0to4] dt + baseline child stunting prevalence[A0to4]* baseline population children[A0to4] | Stunted children population stock with subscripts (i.e. by age group) in increasing order of age | |

| | | | | |
|---|---|---|---|---|
| | | ∫ (( stunted aging[A0to4]- stunted aging[A5to9])+ stunting change[A5to9])- stunted children death rate[A5to9] dt + baseline child stunting prevalence[A5to9]* baseline population children[A5to9] | | |
| | | ∫ (( stunted aging[A5to9]- stunted aging[A10to14])+ stunting change[A10to14])- stunted children death rate[A10to14] dt + baseline child stunting prevalence[A10to14]* baseline population children[A10to14] | | |
| *Flows (N=24)* | | | | |
| adult aging [adult age] | people/Year | adults sb[ adult age]/ adult cohort duration[ adult age] | | |
| adult death rate [adult age] | people/Year | ( adults sb[ adult age]* adult mortality rate[ adult age]) | | |
| adult overweight aging [adult age] | people/Year | Average adult overweight prevalence[ adult age]* adult aging[ adult age] | | |
| adult overweight change [adult age] | people/Year | IF THEN ELSE((adults sb[adult age] > overweight adults[adult age]) ,<br>IF THEN ELSE(overweight change rate>0,<br>(adults sb[adult age] - overweight adults[adult age]) * overweight change rate,<br>overweight adults[adult age] * overweight change rate), 0) | If overweight adults are less than the overall adults, then:<br>If change rate is positive, it means that there is an increase in risk, i.e. people that are not overweight become overweight. If change rate is negative, it means that there is a decrease in risk, i.e. people that are overweight stop being overweight<br>Otherwise (i.e. overweight adults are not less than overall adults) change is zero (as we cannot have more overweight adults than adults overall) | |
| births | people/Year | SUM( adults sb[ adult age!]* fertility rate[ adult age!])* female percentage | | |

| | | | | |
|---|---|---|---|---|
| change in acceptability | servings/Year | IF THEN ELSE (UPEP acceptability <=0,<br>MAX(0,<br>(change in acceptability due to marketing + change in acceptability due to labelling - Change in acceptability because of nutrition education) / Time to change acceptability),<br>IF THEN ELSE(MPF acceptability<=0, MIN(0,<br>(change in acceptability due to marketing + change in acceptability due to labelling - Change in acceptability because of nutrition education) / Time to change acceptability),<br>(change in acceptability due to marketing + change in acceptability due to labelling - Change in acceptability because of nutrition education) / Time to change acceptability)) | Change in acceptability changes based on: (1) industry marketing, (2) labelling policies, (3) nutrition education policies.<br>We use if statements and min/max to ensure that UPEP and MPF acceptability are never below zero. | |
| change in industry desired consumption | servings/Year | ( Industry goal for UPEP consumption- Industry desired UPEP consumption)/ time for industry to achieve goal | | |
| change in MPF consumption | servings/Year | (( MPF acceptability- MPF consumption habit)+ Change in MPF availability+( change in MPF price* elasticity of demand MPF* MPF consumption habit))/ Habit replacement time | Consumption changes according to: 1. Change in acceptability in relation to consumption, 2. Change in availability, 3. Change in price according to the definition of price elasticity, % change in quantity demanded = % change in price * price elasticity of demand | |
| change in UPEP consumption | servings/Year | ( Change in UPEP consumption through acceptability+ Change in UPEP availability+ Change in UPEP consumption through price)/ Habit replacement time | Consumption changes according to: 1. Change in acceptability in relation to consumption, 2. Change in availability, 3. Change in price according to the definition of price elasticity, % change in quantity demanded = % change in price * price elasticity of demand | |
| child aging | people/Year | child aging[child age] = children sb[ child age]/ child cohort duration | | |
| child death rate | people/Year | child death rate[child age] = ( children sb[ child age]* child mortality rate[ child age]) | | |

| | | | | |
|---|---|---|---|---|
| child overweight change [child age] | people/Year | IF THEN ELSE((children sb[child age] > overweight children[child age]) , IF THEN ELSE(overweight change rate>0, (children sb[child age] - overweight children[child age]) * overweight change rate, overweight children[child age] * overweight change rate), 0) | If overweight children are less than the overall children, then:<br>If change rate is positive, it means that there is an increase in risk, i.e. people that are not overweight become overweight. If change rate is negative, it means that there is a decrease in risk, i.e. people that are overweight stop being overweight.<br>Otherwise (i.e. overweight children are not less than overall children, change is zero (as we cannot have more overweight children than children overall) | |
| End of double-duty policies | policy/Year | Active Double-duty policies/ Lifespan of nutrition policies | | |
| End of overnutrition policies | policy/Year | Active overnutrition policies/ Lifespan of nutrition policies | | |
| End of undernutrition policies | policy/Year | Active undernutrition policies/ Lifespan of nutrition policies | | |
| New double-duty policies | policy/Year | ( Double-duty policies/ Time for policy implementation)*STEP( S6,2015) | | |
| New overnutrition policies | policy/Year | Overnutrition policies/ Time for policy implementation | | |
| New undernutrition policies | policy/Year | Undernutrition policies/ Time for policy implementation | | |
| overweight aging [child age] | people/Year | Average child overweight prevalence[ child age]* child aging[ child age] | | |
| overweight births | people/Year | Baseline infant overweight prevalence* births | | |
| Overweight adult death rate [adult age] | people/Year | Average adult overweight prevalence[ adult age]* adult death rate[ adult age] | | |
| Overweight children death rate [child age] | people/Year | Average child overweight prevalence[ child age]* child death rate[ child age] | | |
| Short stature adult death rate [adult age] | people/Year | average short stature prevalence[ adult age]* adult death rate[ adult age] | | |
| short stature aging [adult age] | people/Year | average short stature prevalence[ adult age]* adult aging[ adult age] | | |

| | | | | |
|---|---|---|---|---|
| stunted aging [child age] | people/Year | average stunting prevalence[ child age]* child aging[ child age] | | |
| stunted births [adult age] | people/Year | Baseline low birth weight* births*((1- low birth weight rate[ adult age])+( low birth weight rate[ adult age]* low birth weight and stunting RR)) | | |
| stunted children death rate[child age] | people/Year | average stunting prevalence[ child age]* child death rate[ child age] | | |
| stunting change [child age] | people/Year | IF THEN ELSE((children sb[child age] > stunted children[child age]) ,<br>IF THEN ELSE(stunting change rate>0,<br>(children sb[child age] - stunted children[child age]) * stunting change rate,<br>stunted children[child age] * stunting change rate), 0) | If stunted children are less than the overall children, then:<br>If change rate is positive, it means that there is an increase in risk, i.e. people that are not stunted become stunted. If change rate is negative, it means that there is a decrease in risk, i.e. people that are stunted stop being stunted<br>Otherwise (i.e. stunted children are not less than overall children, change is zero (as we cannot have more stunted children than children overall) | |
| *Smooth variables (N=3)* | | | | |
| Double-duty policies | policy | SMOOTH(( Political will for nutrition policy+( Political will for nutrition policy* DBM gap))/ Political will per policy, Time between political will and action for overnutrition policy) | Double-duty policies are implemented based on the gap between DBM prevalence and goals. The launch of these policies is delayed by the time it takes for political will to become action. They only exist in Scenario 6, for which we assume that total will for nutrition policy is dedicated towards double-duty actions. This can further increase if the gap increases. | |
| Overnutrition policies | policy | SMOOTH( Political will for overnutrition policy/ Political will per policy, Time between political will and action for overnutrition policy) | Overnutrition policies are implemented according to political will for overnutrition action. The launch of these policies is delayed by the time it takes for political will to become action. | |
| Undernutrition policies | policy | SMOOTH( Political will for undernutrition policy/ Political will per policy, Time between political will and action for undernutrition policy) | Undernutrition policies are implemented according to political will for undernutrition action. The launch of these policies is delayed by the time it takes for political will to become action. | |

When the terms [adult age] or [child age] are presented next to the variable name, it means that this variable takes different values per age group. The adult age groups are: 15-24, 25-34, and 35-49 and the child age groups are: 0-4, 5-9, 10-14. The different values are separated by comma in the third column, presented in increasing order of age. The unit Dmnl stands for dimensionless and it is usually used for percentages.

**Supplementary Table S2. Main feedback mechanisms that drive model behaviour**

| Feedback Loop | Feedback Loop name | Feedback Loop description |
|---|---|---|
| R1 | Policy prioritisation and overnutrition policy success | The historic prioritisation of undernutrition policies leads to less political will for overnutrition policy and less overnutrition policy. This leads to more ultra-processed food consumption, as policies that target ultra-processed foods reduce, which leads to higher overweight. This increase in overweight reduces the perceived success of overnutrition policy, further reinforcing the historic prioritisation of undernutrition over overnutrition policy. |
| R2 | Social and economic policy success against undernutrition | Undernutrition policy includes social and economic policies. Thus, an increase in undernutrition policy, leads to an increase in social and economic policies that target stunting, resulting in less stunting. This stunting reduction leads to higher undernutrition policy success, which reinforces the prioritisation of undernutrition over overnutrition policy, resulting in more political will for undernutrition policy and implementation of undernutrition policy. |
| R3 | Food policy success against undernutrition | Undernutrition policy also includes policies that increase consumption of healthy minimally processed foods, through for example food assistance programmes. This increases consumption of healthy food, which can further reduce stunting. Reduction of stunting further leads to more implementation of undernutrition policy, similarly to R2. |
| R4 | Unintended side effect of undernutrition food policy on overweight | Undernutrition policy could also lead to higher ultra-processed food intake, when food offered through food assistance programmes does not comply with relevant regulations and offers ultra-processed foods. This can have an unintended side-effect by further increasing overweight. The increase in overweight further prioritises undernutrition over overnutrition policy, similarly to R1. |
| R5 | Industry response to overnutrition policy through marketing and production | Reduction in ultra-processed foods, for example through overnutrition policy, leads to a reduction in food industry profit. This leads industry to invest further resources to their marketing and/or production and availability of ultra-processed foods, with the goal to increase consumption of ultra-processed foods and thus their profits. |
| B1 | Industry response to overnutrition policy through lobbying | Similarly to R5, reduction of ultra-processed foods and industry profits, leads to industry response by increasing their lobbying against government action for overnutrition policy. Increased lobbying will lead to less overnutrition policy, which resists further reduction of ultra-processed foods and associated industry profits. |

**Supplementary Table S3. Modelling assumptions made under each scenario**

| Scenario | Scenario description | Modelling assumptions |
|---|---|---|
| S1 | Introduction of price policies | The percentage of overnutrition policies that are price policies, e.g. taxes, increase from 0% to 25%. Price policies are assumed to increase price of ultra-processed foods by 15%. |
| S2 | Reduce policy delay, i.e. the time between political will and policy action for both overnutrition and undernutrition | The normal time between political will for overnutrition and undernutrition policy and overnutrition and undernutrition policy action drops from 8 years to 1 year. This does not take into account delay related to industry lobbying for overnutrition policy. |
| S3 | Increase overweight monitoring: Overnutrition policy success is as well monitored as undernutrition policy success | Overnutrition policy success is informed by changes in adult overweight prevalence, in the same way that undernutrition policy success is informed by under-5 stunting prevalence. This is different from the baseline scenario, where overnutrition policy success was diluted, by assuming that only 50% of changes in overweight prevalence inform overnutrition policy success, compared to undernutrition policy success, which was informed by 100% of changes in under-5 stunting prevalence. |
| S4 | Regulate quality of foods available through undernutrition policies: No ultra-processed foods can be provided through undernutrition policies, such as food assistance programmes | The amount of ultra-processed foods provided through food assistance policies drops to 0%. At baseline, the percentage of ultra-processed foods provided through food assistance programmes was assumed to be equal to the percentage of ultra-processed foods of the overall diet of the population, estimated using the ultra-processed and minimally processed food consumption stocks in the model. |
| S5 | Regulate industry lobbying: Industry plays no role in the overnutrition policy making process | The delay between political will for overnutrition policy and overnutrition policy action due to lobbying drops from 6 years to 0 years. |
| S6 | Double-duty policies informed by malnutrition in all its forms progress | Nutrition policy does not rely on prioritisation of undernutrition over overnutrition. There are no more overnutrition and undernutrition policies, but only double-duty policies. Double-duty policies are informed by political will for nutrition policy, which is not anymore split between political will for overnutrition and undernutrition but can change according to the difference between adult overweight prevalence and under-5 stunting prevalence and pre-determined goals for these two metrics. This means that political will for nutrition policy can increase until both of these goals are met. |

All scenarios are introduced in 2015. Each Scenario includes all the previous scenarios

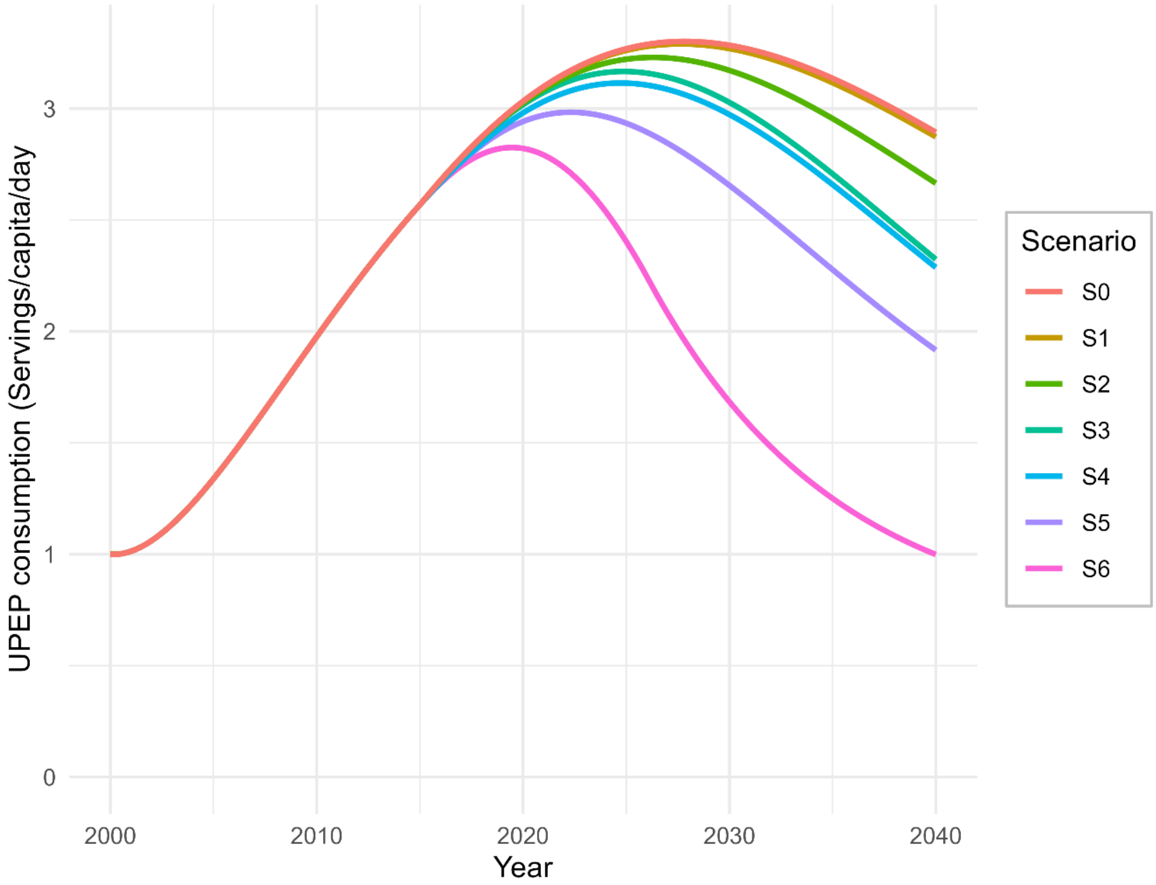


**Supplementary Figure S1. Behaviour over-time graph for ultra-processed food consumption, under different scenarios**

**References**


1 Sterman JD. Business Dynamics Systems Thinking and Modeling for a Complex World. 2000.

2 Epstein JM. Why Model? *Journal of Artificial Societies and Social Simulation* 2008; **11**: 4–9.

3 Forrester JW. Urban Dynamics. Portland, Or: Productivity Press, 1969.

4 Norström P. Engineers' non-scientific models in technology education. *Int J Technol Des Educ* 2013. DOI:10.1007/s10798-011-9184-2.

5 Sterman J. Business dynamics. McGraw-Hill, Inc., 2000.

6 Dirección Técnica de Demografía e Indicadores Sociales del Instituto Nacional de Estadística e Informática (INEI). Perú: Situación y Perspectivas de la Mortalidad por Sexo y Grupos de Edad, Nacional y por Departamentos, 1990-2025. Lima, 2010 http://proyectos.inei.gob.pe/web/biblioineipub/bancopub/Est/Lib0901/index.htm.

7 Instituto Nacional de Estadística e Informática. Series anuales de indicadores principales de la ENDES 1986 - 2019. 2020 https://proyectos.inei.gob.pe/endes/series.asp (accessed April 27, 2021).

8 Instituto Nacional de Estadística e Informática (INEI). Encuesta Demográfica y de Salud Familiar (ENDES). 2000.

9 NCD-RisC. Children & Adolescent BMI. https://ncdrisc.org/data-downloads-adiposity-ado.html (accessed May 5, 2024).

10 University of Oxford. Young Lives: an International Study of Childhood Poverty. [data series]. 2019.

11 Carrillo-Larco RM, Cajachagua-Torres KN, Guzman-Vilca WC, Quezada-Pinedo HG, Tarazona-Meza C, Huicho L. National and subnational trends of birthweight in Peru: Pooled analysis of 2,927,761 births between 2012 and 2019 from the national birth registry. *The Lancet Regional Health - Americas* 2021; **1**: 100017.

12 Blencowe H, Krasevec J, de Onis M, *et al.* National, regional, and worldwide estimates of low birthweight in 2015, with trends from 2000: a systematic analysis. *Lancet Glob Health* 2019; **7**: e849–60.

13 Instituto Nacional de Estadística e Informática. Perú: Estimaciones y Proyecciones de la Población Nacional, Por Año Calendario y Edad Simple, 1950-2050. Lima, 2019 https://www.inei.gob.pe/media/MenuRecursivo/publicaciones_digitales/Est/Lib1681/.

14 Sadeghirad B, Duhaney T, Motaghipisheh S, Campbell NRC, Johnston BC. Influence of unhealthy food and beverage marketing on children's dietary intake and preference: a systematic review and meta-analysis of randomized trials. *Obesity Reviews* 2016; **17**: 945–59.

15 Huicho L, Vidal-Cárdenas E, Akseer N, *et al.* Drivers of stunting reduction in Peru: A country case study. *American Journal of Clinical Nutrition* 2020; **112**: 816S-829S.

16 Nisbett N, Salm L, Roelen K, *et al.* Social Protection Pathways to Nutrition. 2023.

17 Askari M, Heshmati J, Shahinfar H, Tripathi N, Daneshzad E. Ultra-processed food and the risk of overweight and obesity: a systematic review and meta-analysis of observational studies. *International Journal of Obesity 2020 44:10* 2020; **44**: 2080–91.

18 Taillie LS, Hall MG, Gómez LF, *et al.* Designing an effective front-of-package warning label for food and drinks high in added sugar, sodium, or saturated fat in colombia: An online experiment. *Nutrients* 2020; **12**: 1–20.

19 Muhammad A, D'Souza A, Meade B, Micha R, Mozaffarian D. How income and food prices influence global dietary intakes by age and sex: evidence from 164 countries. *BMJ Glob Health* 2017; **2**: e000184.

20 INEI. Encuesta Demográfica y de Salud Familiar ENDES 2020. 2021.

21 Pan American Health Organization. Ultra-processed food and drink products in Latin America: Trends, impact on obesity, policy implications. US1.1. 2015; : 1–58.

22 Instituto Nacional de Estadística e Informática (INEI). Encuesta Nacional de Hogares (ENAHO). 2000.

23 Global Dietary Database. 2000.

24 Meza-Hernández M, Villarreal-Zegarra D, Saavedra-Garcia L. Nutritional quality of food and beverages offered in supermarkets of lima according to the peruvian law of healthy eating. *Nutrients* 2020; **12**. DOI:10.3390/nu12051508.

25 Mejía Acosta A, Haddad L. The politics of success in the fight against malnutrition in Peru. *Food Policy* 2014; **44**: 26–35.

26 Christian P, Lee SE, Angel MD, *et al.* Risk of childhood undernutrition related to small-for-gestational age and preterm birth in low- and middle-income countries. *Int J Epidemiol* 2013; **42**: 1340–55.

27 Kozuki N, Katz J, Lee ACC, *et al.* Short maternal stature increases risk of small for-gestational-age and preterm births in lowand middle-income countries: Individual participant data meta-analysis and population attributable fraction. *Journal of Nutrition* 2015; **145**: 2542–50.

28 World Health Organization. Global Targets 2025: To improve maternal, infant and young child nutrition. .

29 Saavedra-Garcia L, Taboada-Ramirez X, Hernández-Vásquez A, Diez-Canseco F. Marketing techniques, health, and nutritional claims on processed foods and beverages before and after the implementation of mandatory front-of-package warning labels in Peru. *Front Nutr* 2022; **9**. DOI:10.3389/fnut.2022.1004106.

30 Huicho L, Segura ER, Huayanay-Espinoza CA, *et al.* Child health and nutrition in Peru within an antipoverty political agenda: A Countdown to 2015 country case study. *Lancet Glob Health* 2016; **4**: e414–26.
