# Supplementary material for: Actions against the double burden of malnutrition in Peru: a community-informed system dynamics model
Source: Lancet Reg Health Am. 2025 Apr 21;46:101102. doi: 10.1016/j.lana.2025.101102 (PMC12052979; doi:10.1016/j.lana.2025.101102)
Supplement: Abstract in Spanish [file mmc2.pdf]

Editorial disclaimer: This translation in Spanish was submitted by the authors and we reproduce it as supplied. It has not been peer-reviewed. Our editorial processes have only been applied to the original abstract in English, which should serve as a reference for this manuscript.

RESUMEN

Antecedentes

El progreso peruano en la reducción de la desnutrición se ha estancado desde el 2018, mientras, a la vez, enfrenta niveles crecientes de sobrenutrición, llevando a doble carga de malnutrición. Sin embargo, este cambio en la carga nutricional no se refleja en la agenda de políticas nutricionales del país. Este estudio tiene como objetivo identificar puntos de apalancamiento para tomar acciones contra la doble carga de malnutrición a nivel poblacional en el Perú.

Métodos

Se desarrolló un modelo de dinámica de sistemas que simula cambios en el sobrepeso y desnutrición a lo largo del tiempo en Perú a través de cambios en los impulsores del sistema alimentario. El modelo fue informado conceptualmente por formuladores de políticas, profesionales y miembros de la comunidad en Perú y utilizó datos cuantitativos y cualitativos de fuentes secundarias y literatura publicada.

Resultados

El modelo indicó que varias políticas de sobrenutrición, incluidas las políticas dirigidas a la disponibilidad y asequibilidad de los alimentos, pueden desacelerar, pero no detener el aumento del sobrepeso en el país, principalmente debido a la resistencia de la industria. Sin embargo, a largo plazo, la reasignación de recursos hacia políticas de sobrenutrición puede obstaculizar involuntariamente el progreso hacia la reducción de la desnutrición. La transformación de la gobernanza de las políticas nutricionales, desde políticas aisladas contra la sobrenutrición y la desnutrición hacia un marco de políticas común contra la doble carga de la malnutrición, fue el único escenario modelado que detuvo el aumento de la sobrenutrición, al tiempo que mantuvo al Perú en el camino hacia el logro de sus objetivos de retraso del crecimiento.

Interpretación

La transición desde el panorama de políticas que se centran en resultados nutricionales aislados hacia acciones sinérgicas que aborden la malnutrición en todas sus formas es una solución a largo plazo para alcanzar los objetivos mundiales en nutrición. Este proceso de transición en las políticas es especialmente importante en países de ingresos bajos y medios como el Perú, afectados por la doble carga de la malnutrición.

Financiamiento

Este estudio fue financiado con una subvención de investigación del Consejo de Investigación en Biotecnología y Ciencias Biológicas (BBSRC de sus siglas en inglés) (referencia de subvención: BB/T009004/1).
